# Supplementary figures and images for: B cell receptor-induced IL-10 production from neonatal mouse CD19+CD43- cells depends on STAT5-mediated IL-6 secretion
Source: eLife. 2023 Feb 3;12:e83561. doi: 10.7554/eLife.83561 (PMC9934864; doi:10.7554/eLife.83561)

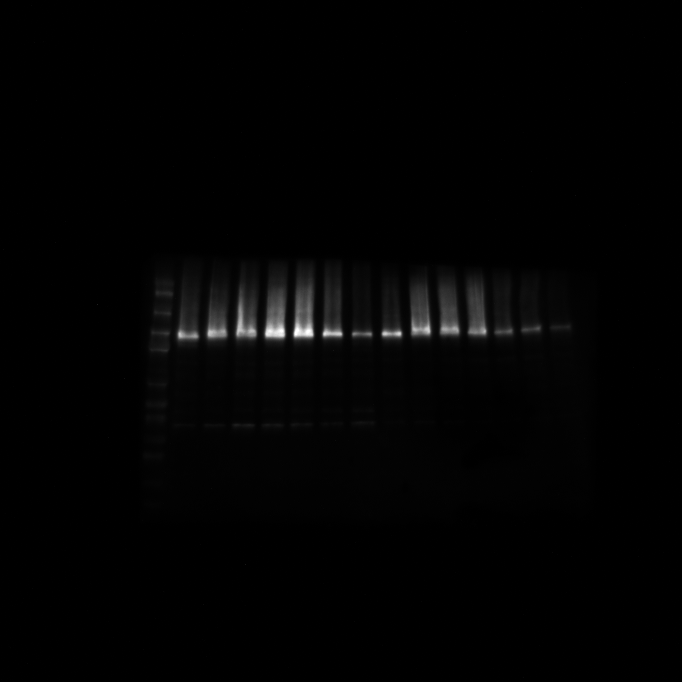

Supplement: Figure 2—source data 1. [file elife-83561-fig2-data1.zip › Figure_2-source_data_1_Figure_2E_total_STAT5.tif]

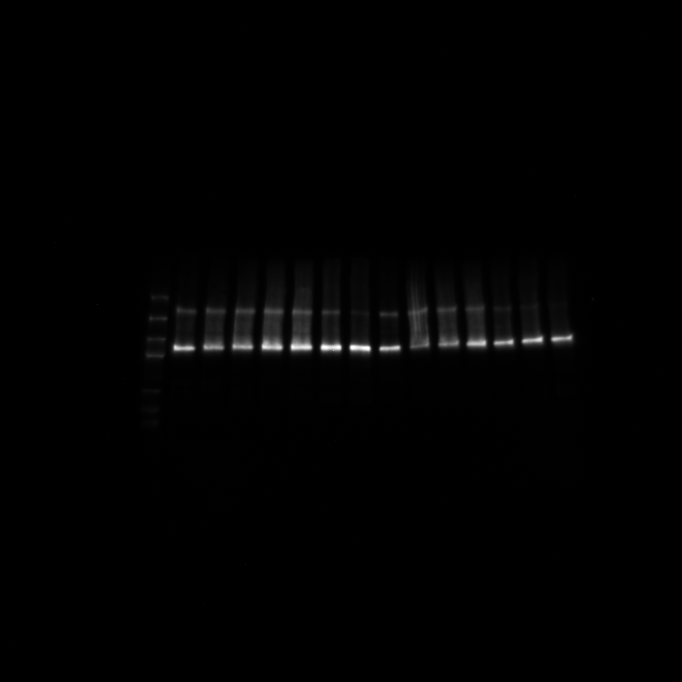

Supplement: Figure 2—source data 1. [file elife-83561-fig2-data1.zip › Figure_2-source_data_1_Figure_2E_total_STAT3.tif]

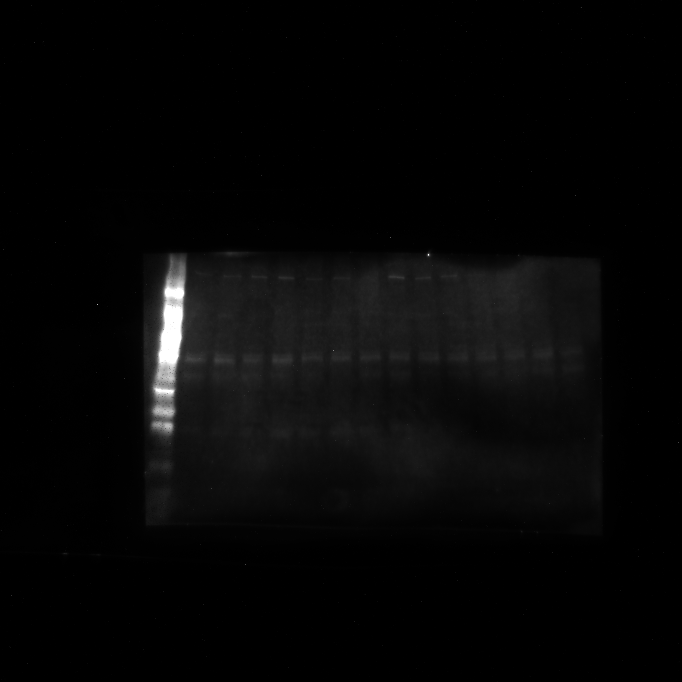

Supplement: Figure 2—source data 1. [file elife-83561-fig2-data1.zip › Figure_2-source_data_1_Figure_2E_total_Stat1.tif]

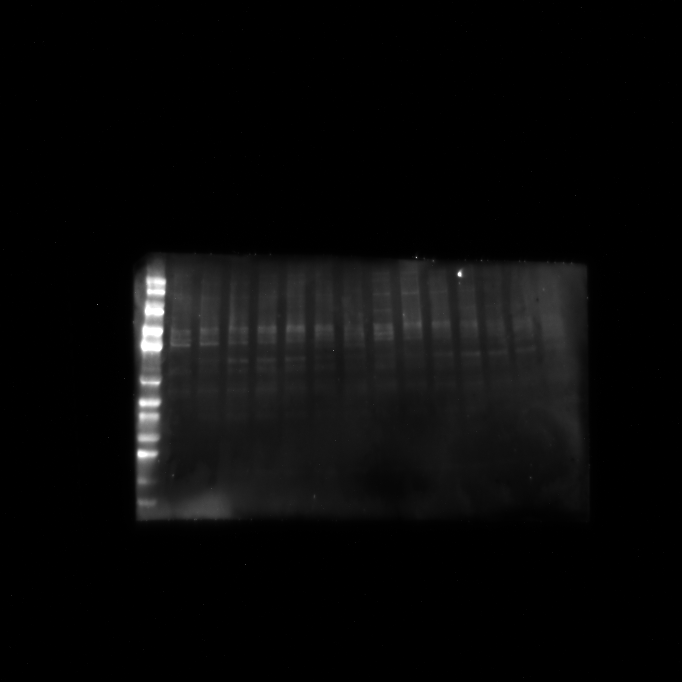

Supplement: Figure 2—source data 1. [file elife-83561-fig2-data1.zip › Figure_2-source_data_1_Figure_2E_p-STAT5.tif]

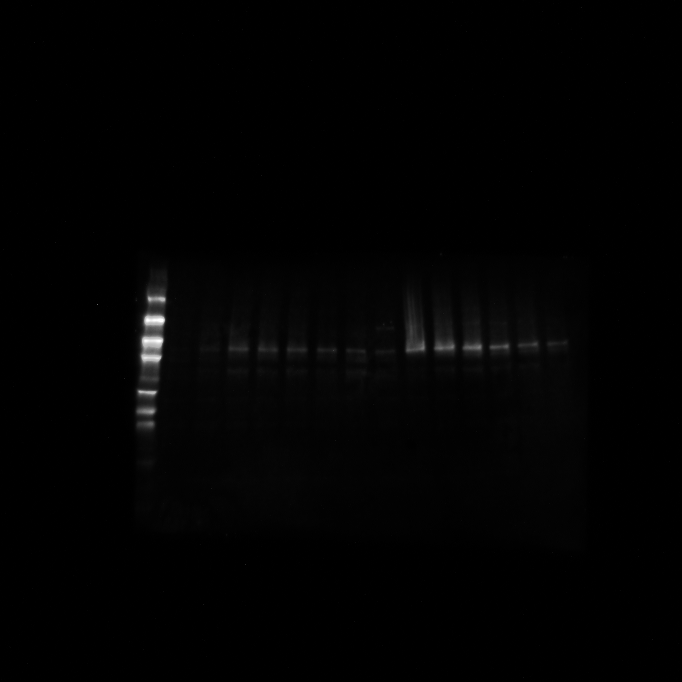

Supplement: Figure 2—source data 1. [file elife-83561-fig2-data1.zip › Figure_2-source_data_1_Figure_2E_p-STAT3.tif]

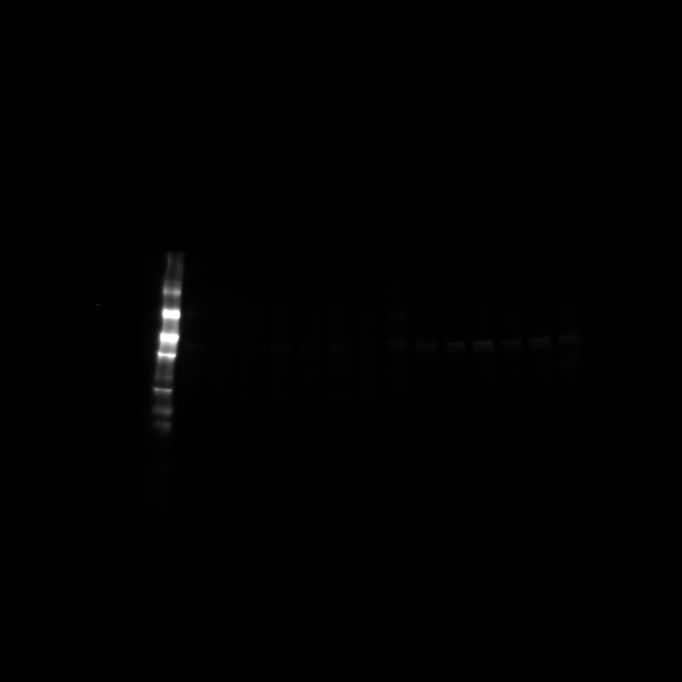

Supplement: Figure 2—source data 1. [file elife-83561-fig2-data1.zip › Figure_2-source_data_1_Figure_2E_p-Stat1.tif]

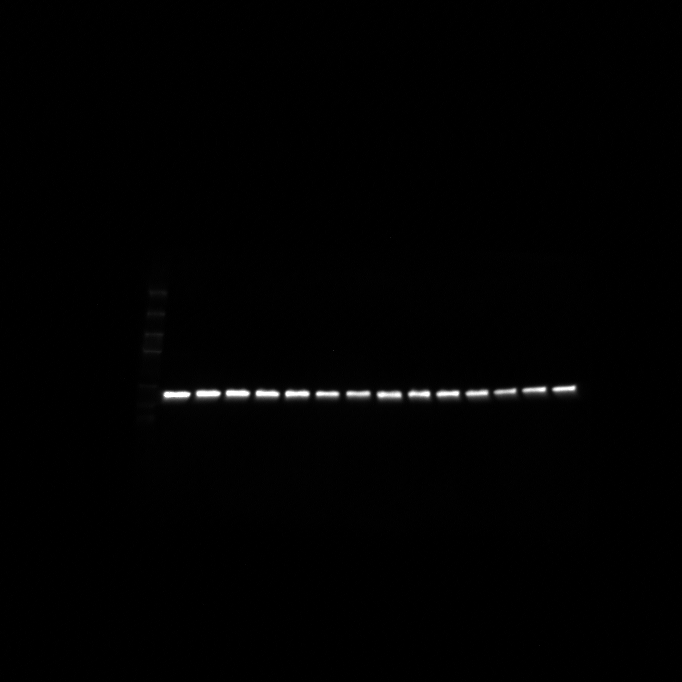

Supplement: Figure 2—source data 1. [file elife-83561-fig2-data1.zip › Figure_2-source_data_1_Figure_2E_b-actin.tif]

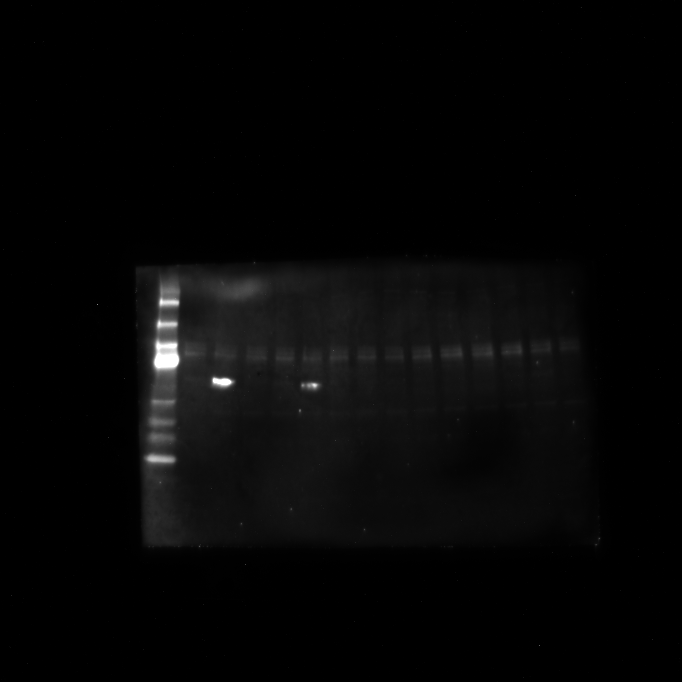

Supplement: Figure 2—source data 1. [file elife-83561-fig2-data1.zip › Figure_2-source_data_1_Figure_2D_total_STAT5.tif]

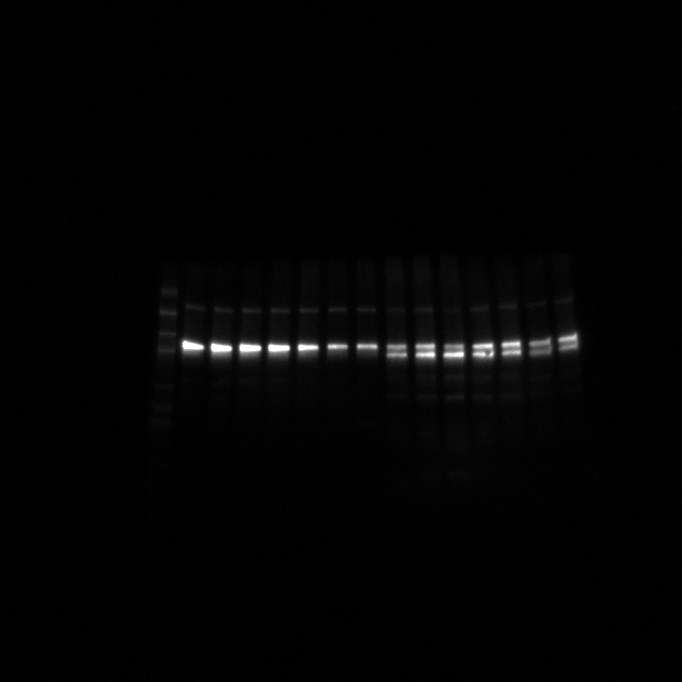

Supplement: Figure 2—source data 1. [file elife-83561-fig2-data1.zip › Figure_2-source_data_1_Figure_2D_total_Stat3.tif]

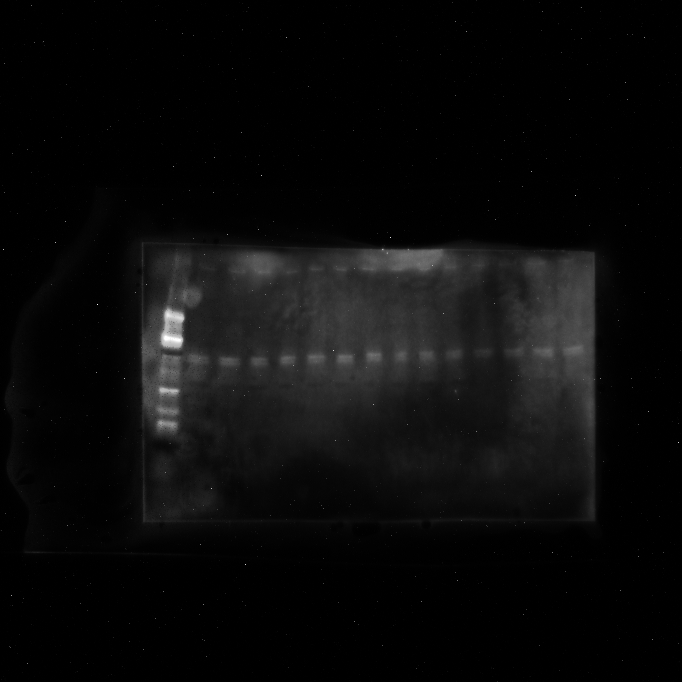

Supplement: Figure 2—source data 1. [file elife-83561-fig2-data1.zip › Figure_2-source_data_1_Figure_2D_total_Stat1.tif]

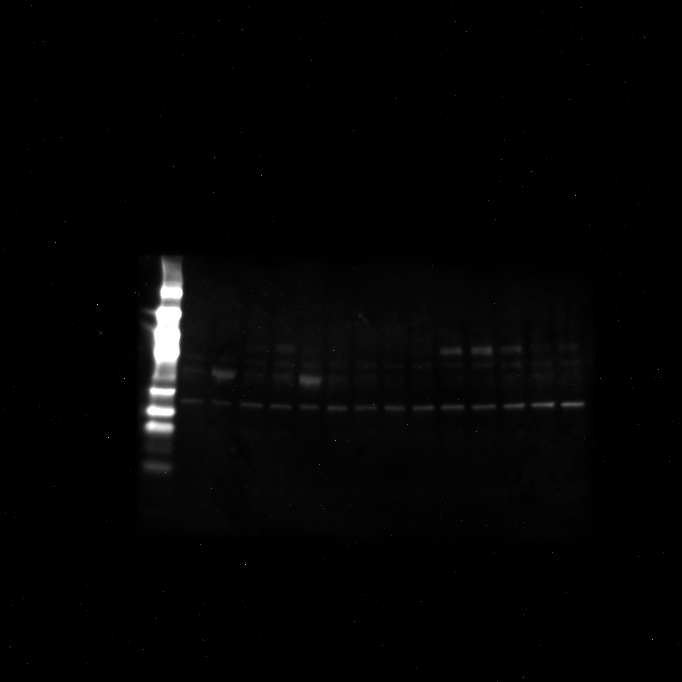

Supplement: Figure 2—source data 1. [file elife-83561-fig2-data1.zip › Figure_2-source_data_1_Figure_2D_p-STAT5.tif]

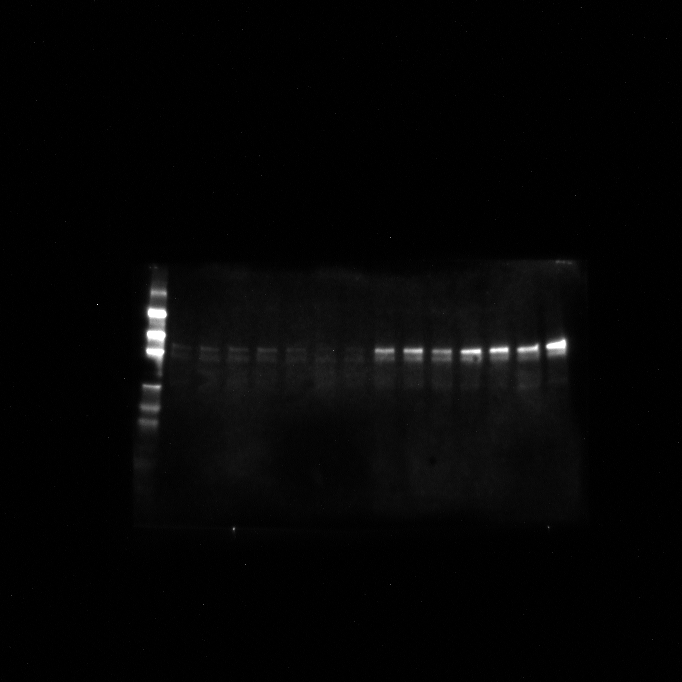

Supplement: Figure 2—source data 1. [file elife-83561-fig2-data1.zip › Figure_2-source_data_1_Figure_2D_p-Stat3.tif]

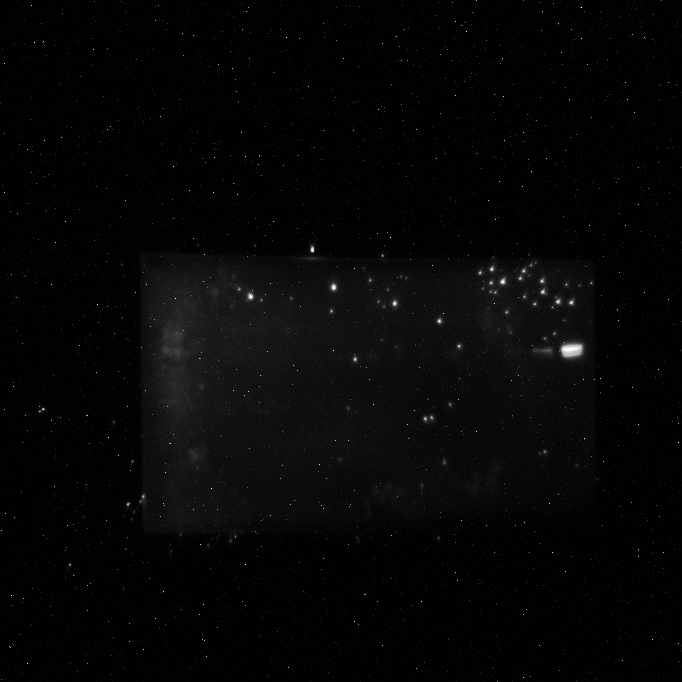

Supplement: Figure 2—source data 1. [file elife-83561-fig2-data1.zip › Figure_2-source_data_1_Figure_2D_p-Stat1.tif]

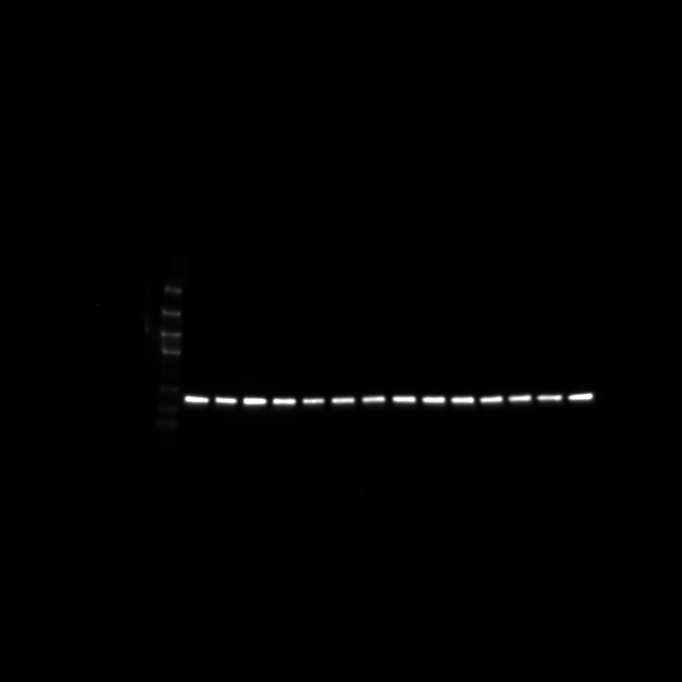

Supplement: Figure 2—source data 1. [file elife-83561-fig2-data1.zip › Figure_2-source_data_1_Figure_2D_b-actin.tif]

# Figure 2-source data 1

Figure 2D

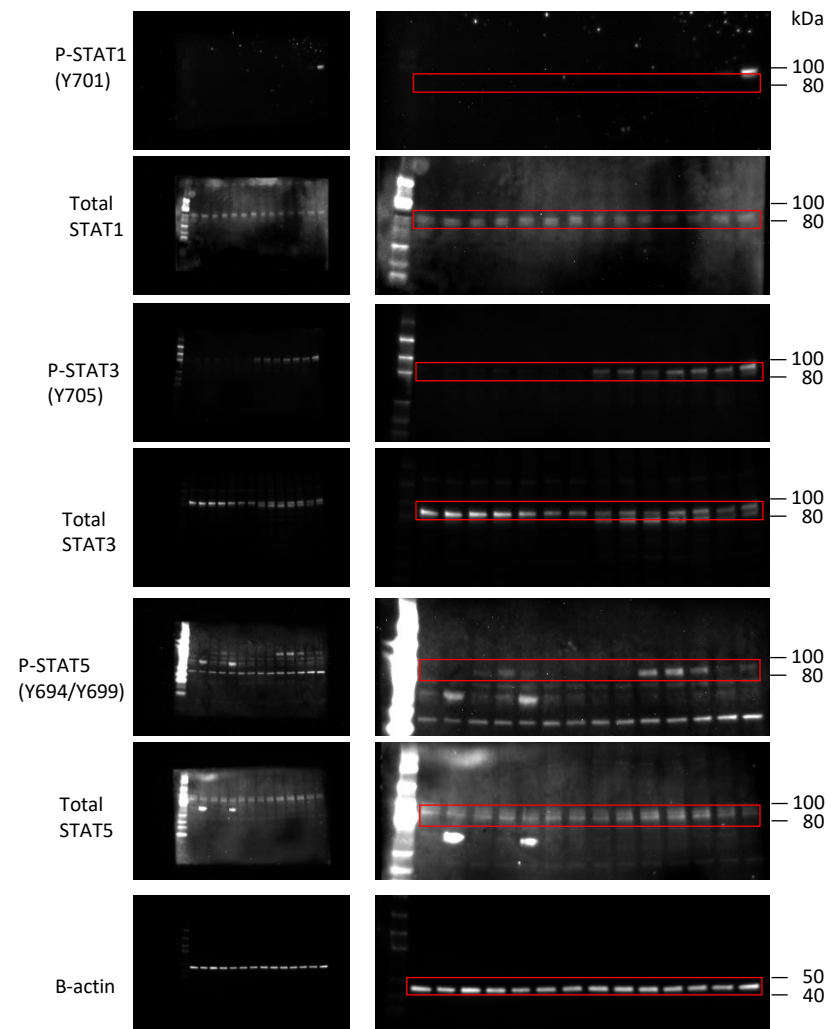

Figure 2E

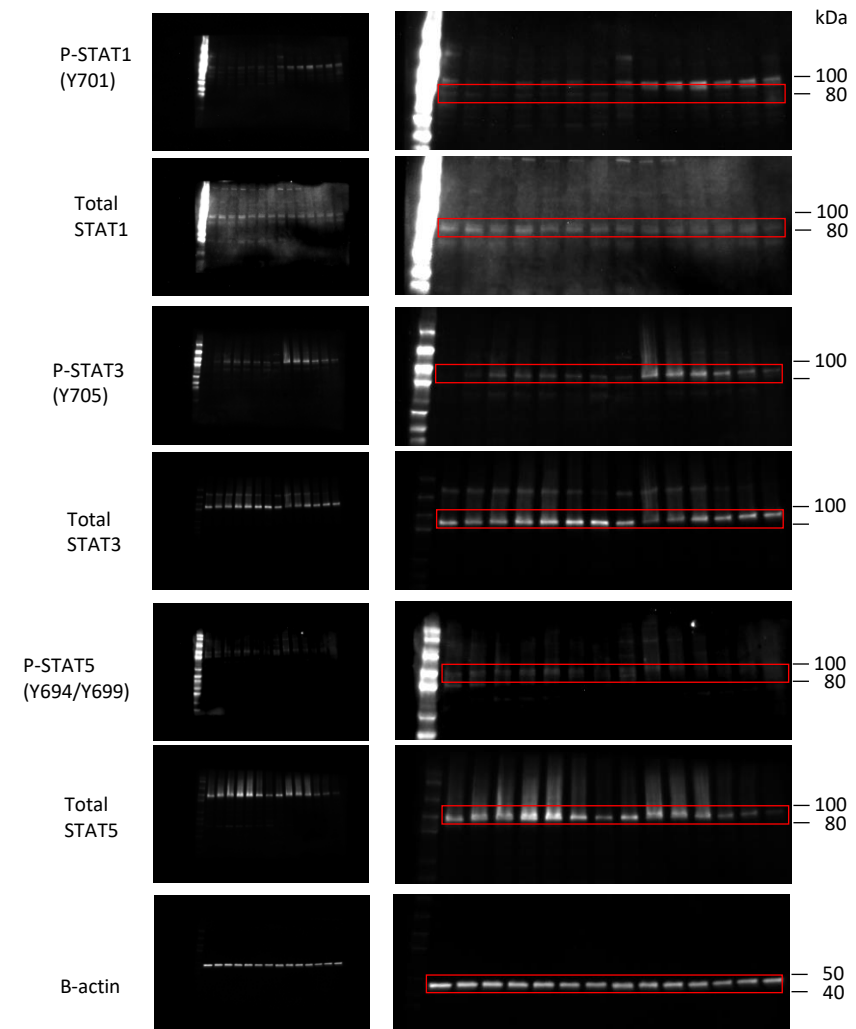

Supplement: Figure 2—source data 2. [file elife-83561-fig2-data2.pdf]

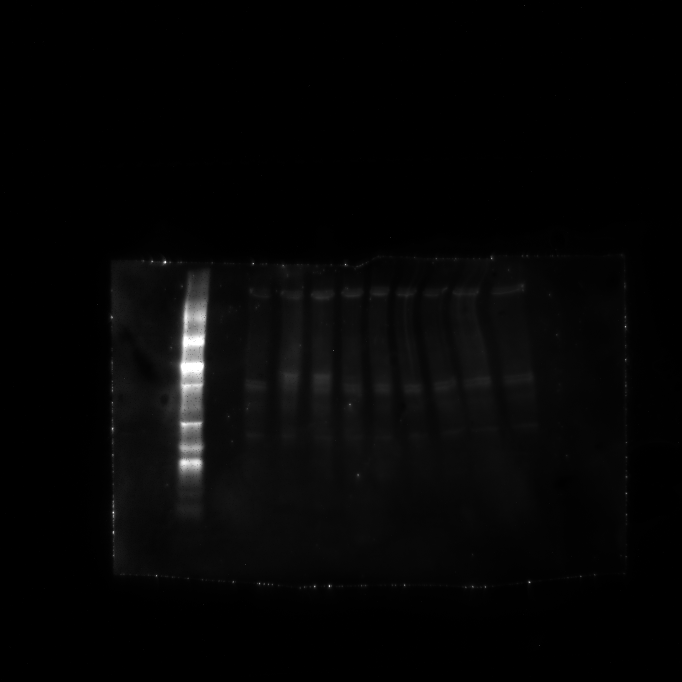

Supplement: Figure 2—figure supplement 2—source data 1. [file elife-83561-fig2-figsupp2-data1.zip › Figure_2-figure_supplement_2-source_data_1_total_STAT1.tif]

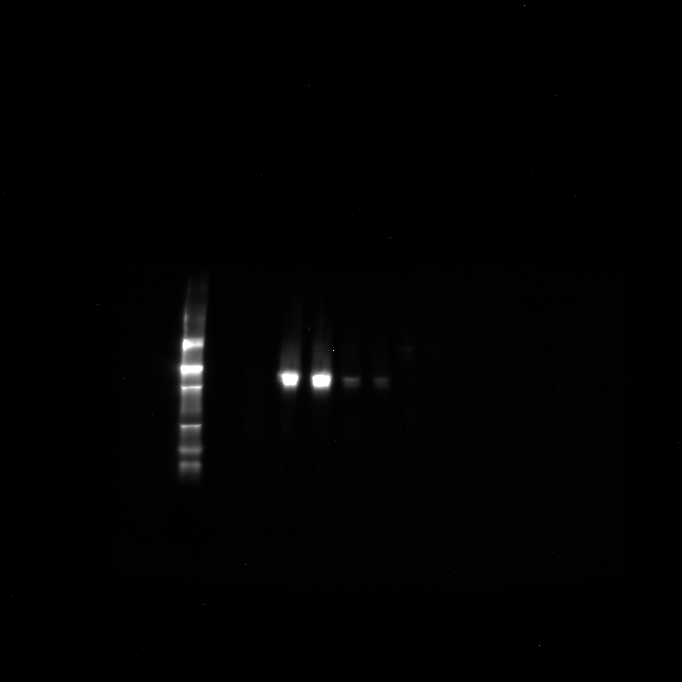

Supplement: Figure 2—figure supplement 2—source data 1. [file elife-83561-fig2-figsupp2-data1.zip › Figure_2-figure_supplement_2-source_data_1_p-STAT1.tif]

**Figure 2-figure supplement 2-source data 1**

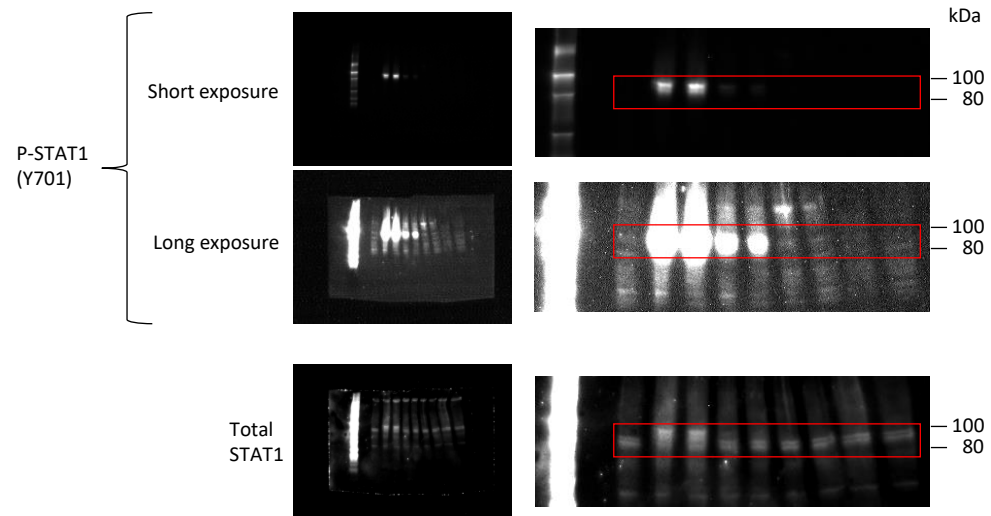

Supplement: Figure 2—figure supplement 2—source data 2. [file elife-83561-fig2-figsupp2-data2.pdf]

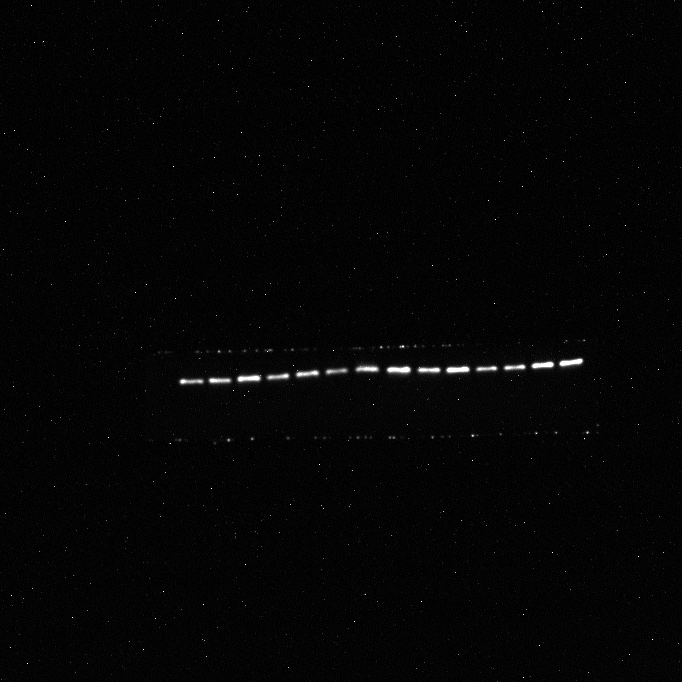

Supplement: Figure 2—figure supplement 3—source data 1. [file elife-83561-fig2-figsupp3-data1.zip › Figure_2-figure_supplement_3-source_data_1_total_p38.tif]

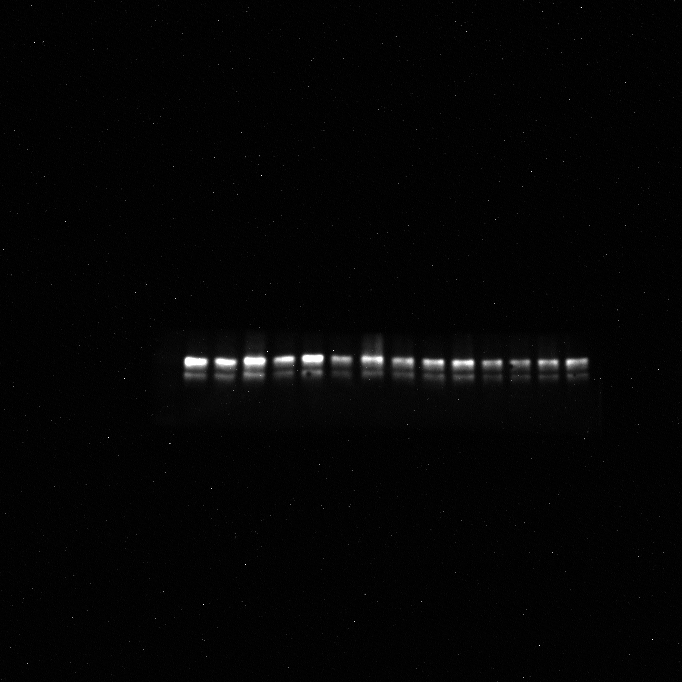

Supplement: Figure 2—figure supplement 3—source data 1. [file elife-83561-fig2-figsupp3-data1.zip › Figure_2-figure_supplement_3-source_data_1_total_JNK.tif]

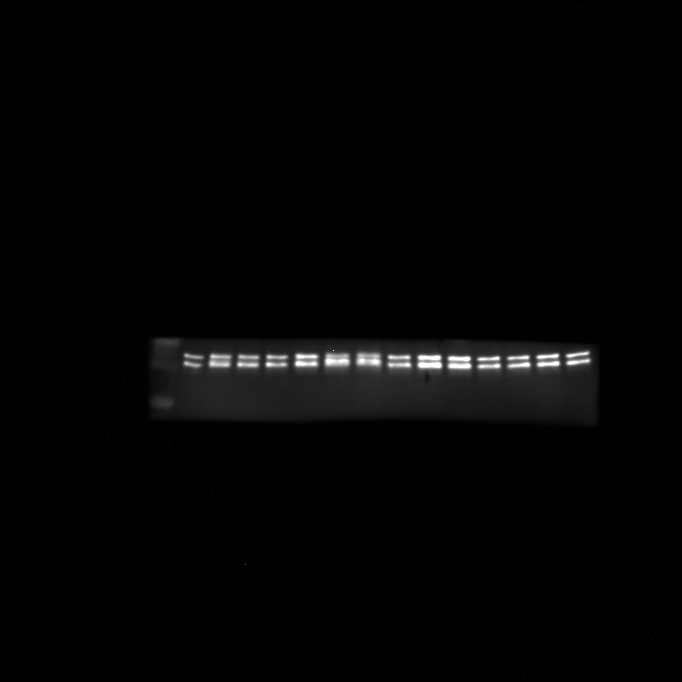

Supplement: Figure 2—figure supplement 3—source data 1. [file elife-83561-fig2-figsupp3-data1.zip › Figure_2-figure_supplement_3-source_data_1_total_Erk.tif]

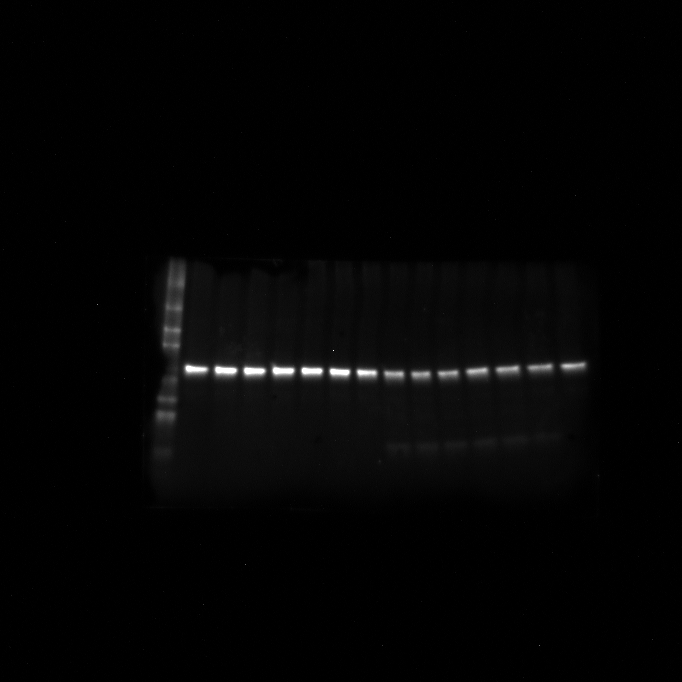

Supplement: Figure 2—figure supplement 3—source data 1. [file elife-83561-fig2-figsupp3-data1.zip › Figure_2-figure_supplement_3-source_data_1_pan_Akt.tif]

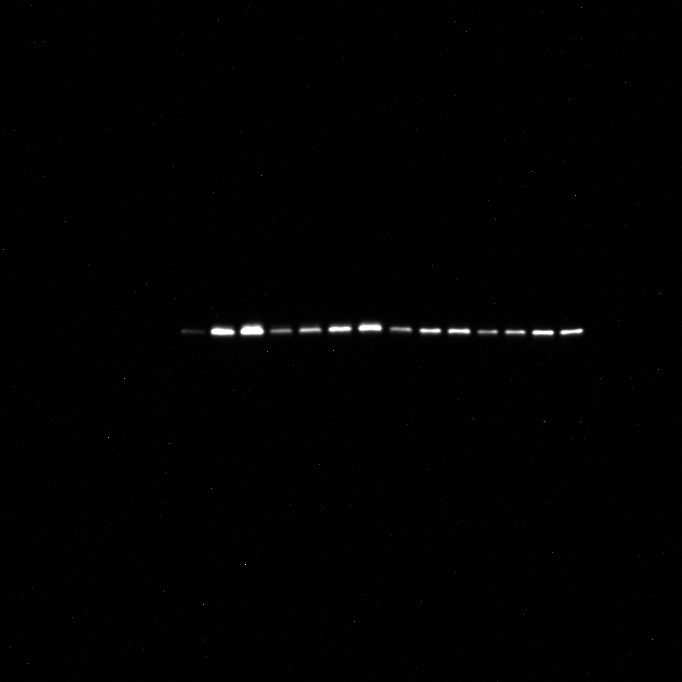

Supplement: Figure 2—figure supplement 3—source data 1. [file elife-83561-fig2-figsupp3-data1.zip › Figure_2-figure_supplement_3-source_data_1_p-p38.tif]

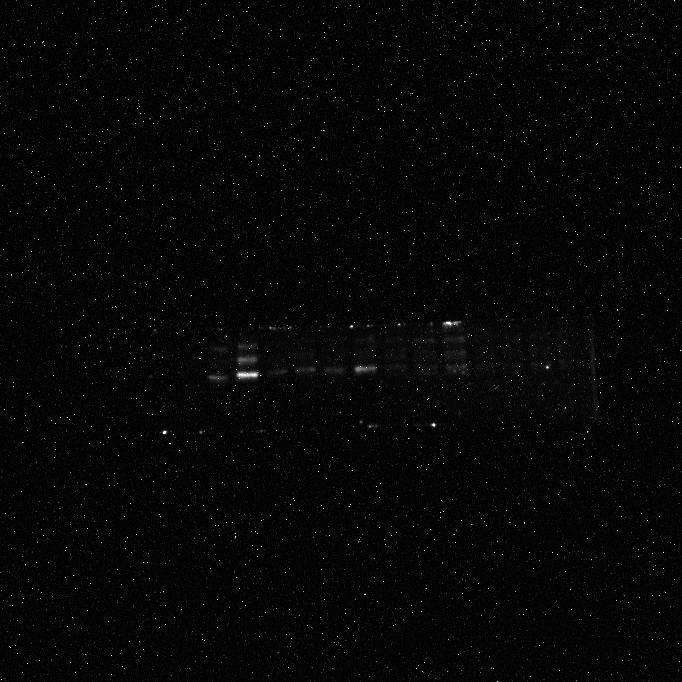

Supplement: Figure 2—figure supplement 3—source data 1. [file elife-83561-fig2-figsupp3-data1.zip › Figure_2-figure_supplement_3-source_data_1_p-JNK.tif]

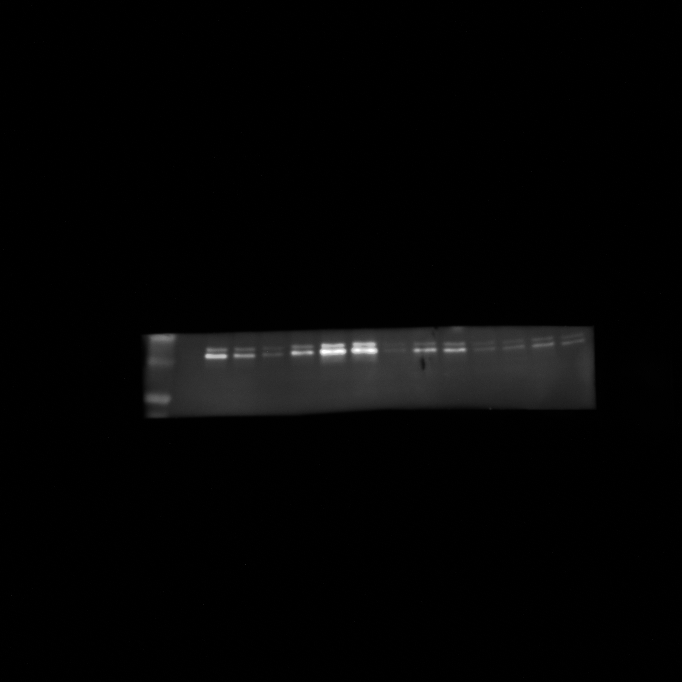

Supplement: Figure 2—figure supplement 3—source data 1. [file elife-83561-fig2-figsupp3-data1.zip › Figure_2-figure_supplement_3-source_data_1_p-Erk.tif]

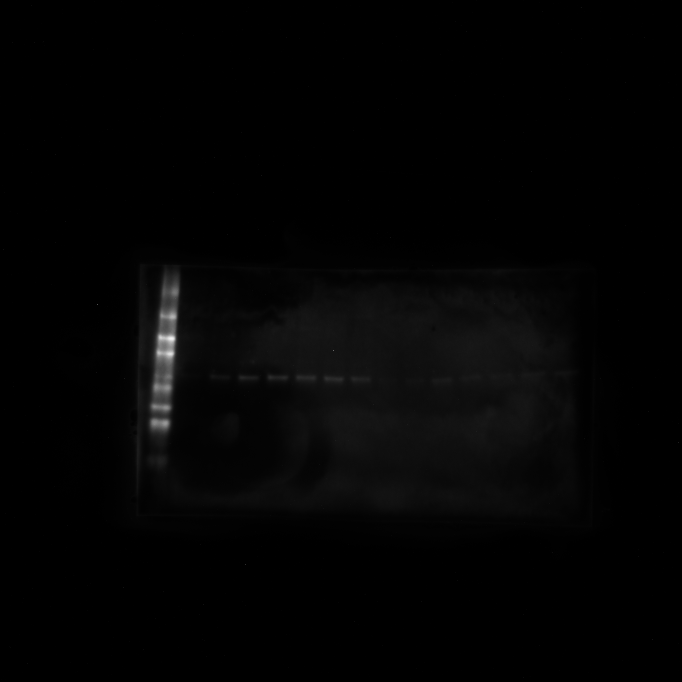

Supplement: Figure 2—figure supplement 3—source data 1. [file elife-83561-fig2-figsupp3-data1.zip › Figure_2-figure_supplement_3-source_data_1_p-Akt.tif]

**Figure 2-figure supplement 3-source data 1**

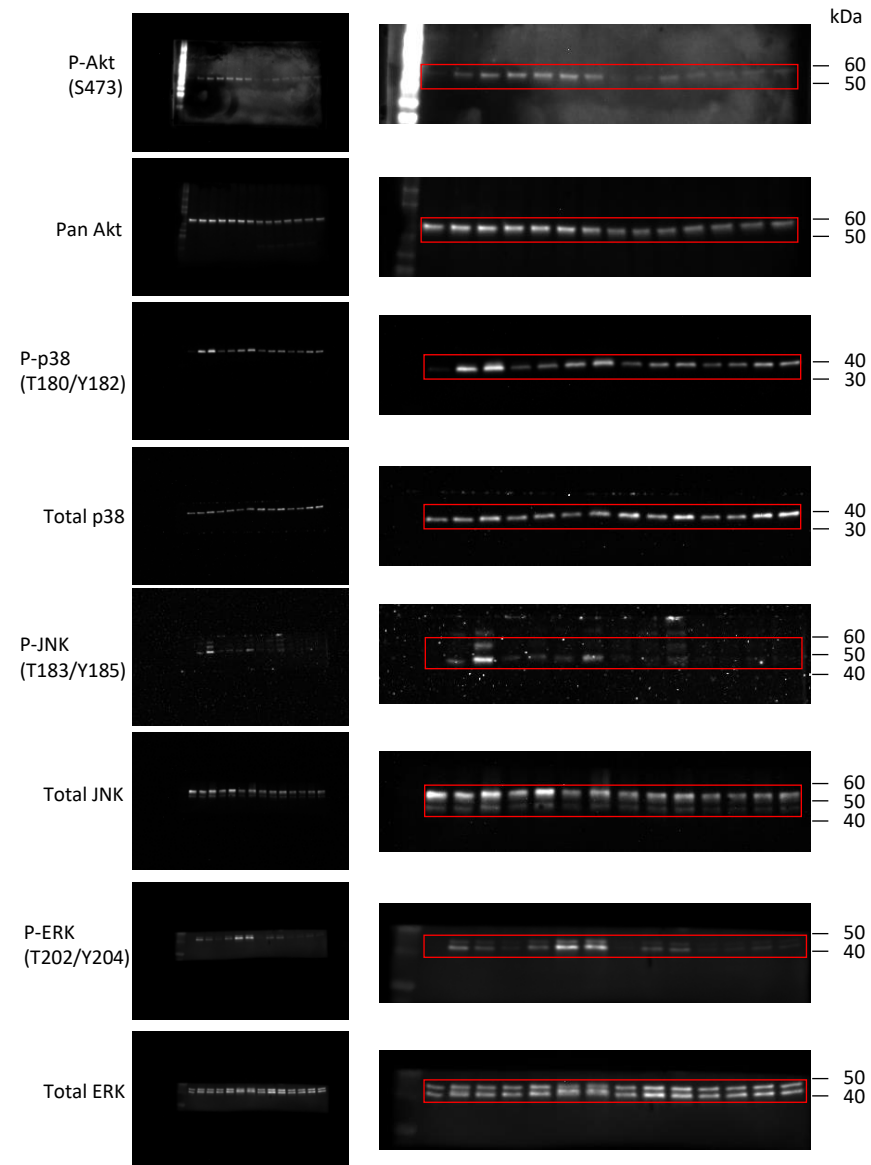

Supplement: Figure 2—figure supplement 3—source data 2. [file elife-83561-fig2-figsupp3-data2.pdf]

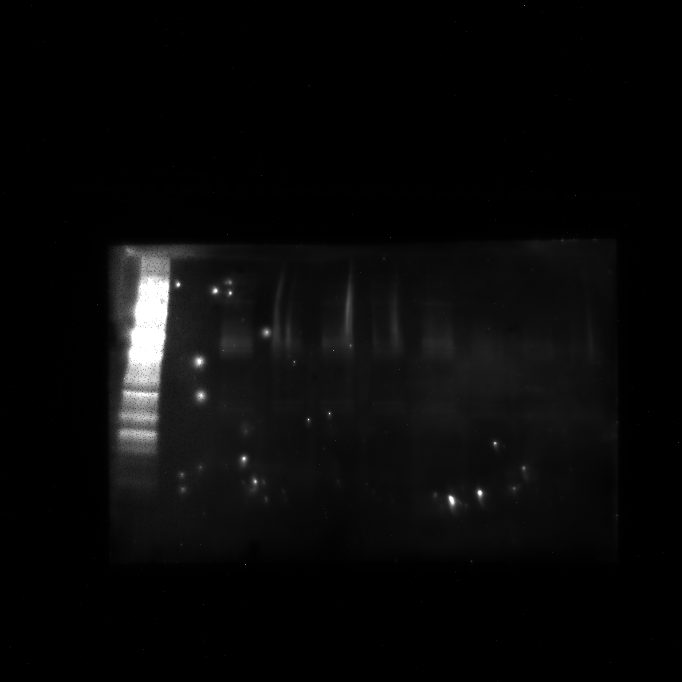

Supplement: Figure 3—source data 1. [file elife-83561-fig3-data1.zip › Figure_3-source_data_1_Figure_3I_STAT5.tif]

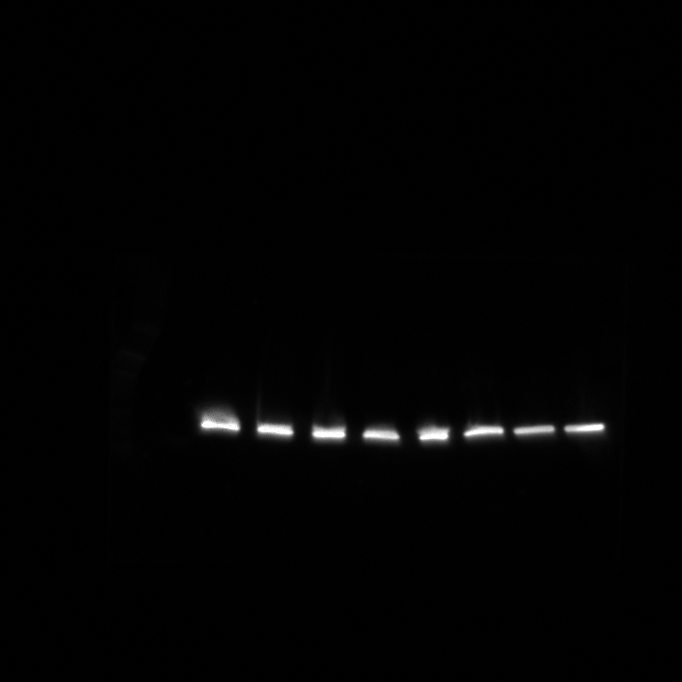

Supplement: Figure 3—source data 1. [file elife-83561-fig3-data1.zip › Figure_3-source_data_1_Figure_3I_B-actin.tif]

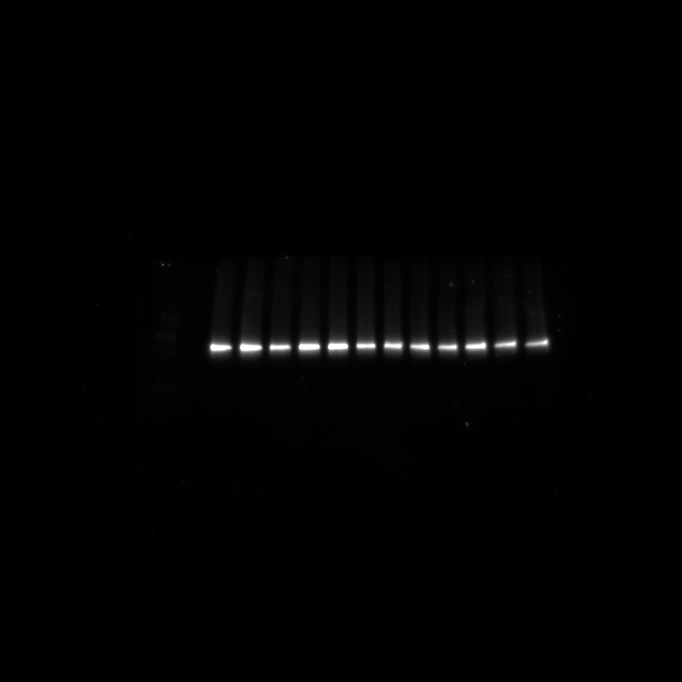

Supplement: Figure 3—source data 1. [file elife-83561-fig3-data1.zip › Figure_3-source_data_1_Figure_3D_total_STAT3.tif]

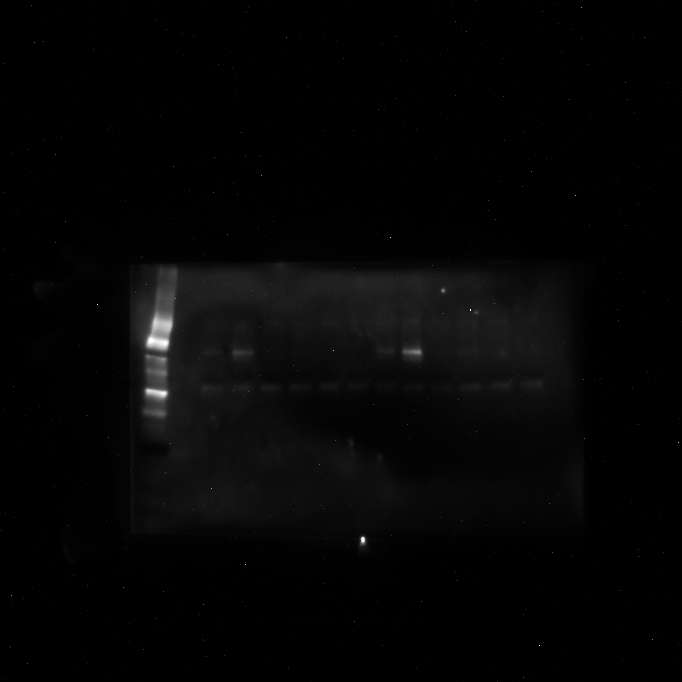

Supplement: Figure 3—source data 1. [file elife-83561-fig3-data1.zip › Figure_3-source_data_1_Figure_3D_p-STAT3.tif]

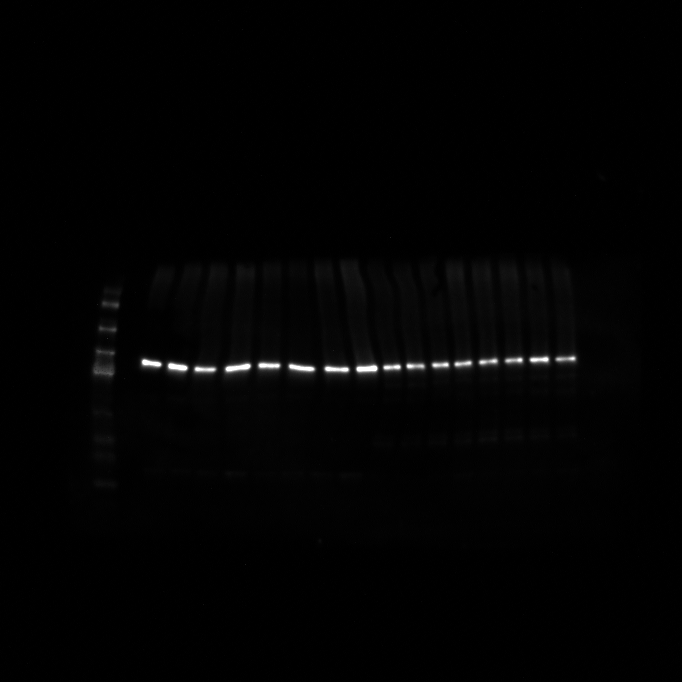

Supplement: Figure 3—source data 1. [file elife-83561-fig3-data1.zip › Figure_3-source_data_1_Figure_3C_total_STAT5.tif]

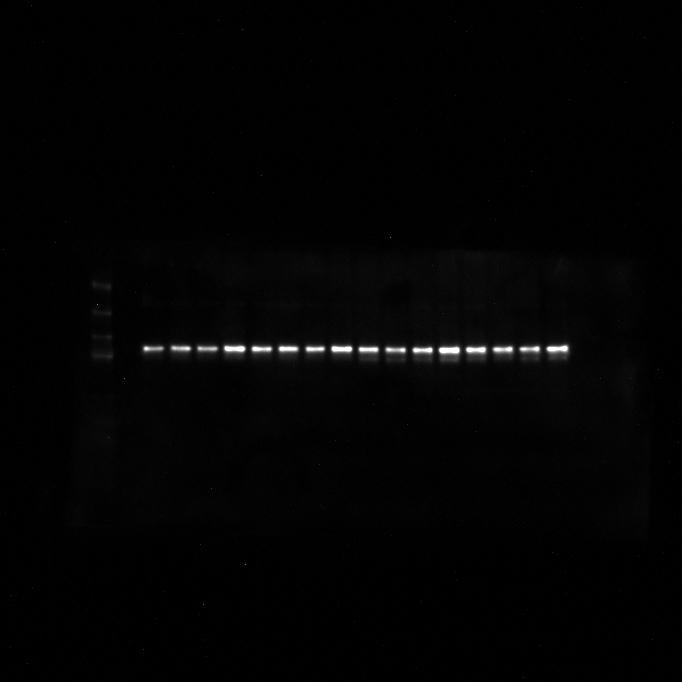

Supplement: Figure 3—source data 1. [file elife-83561-fig3-data1.zip › Figure_3-source_data_1_Figure_3C_total_STAT3.tif]

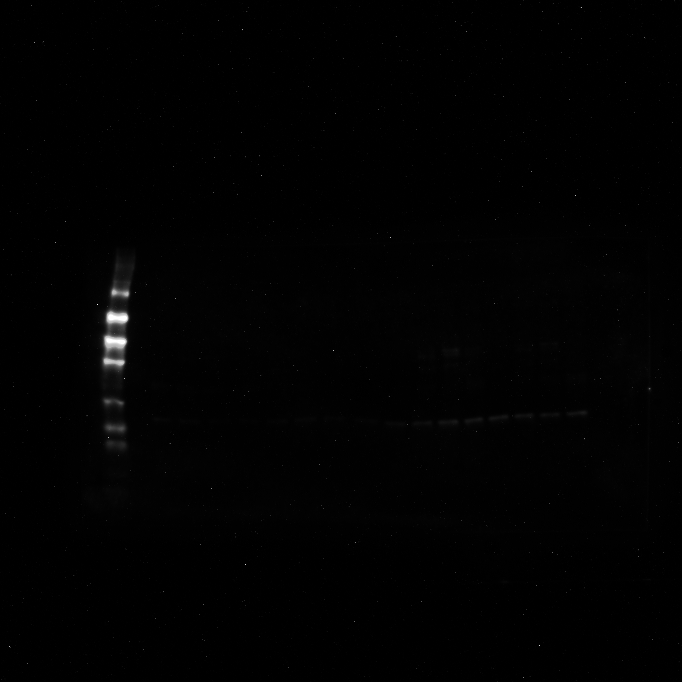

Supplement: Figure 3—source data 1. [file elife-83561-fig3-data1.zip › Figure_3-source_data_1_Figure_3C_p-STAT5.tif]

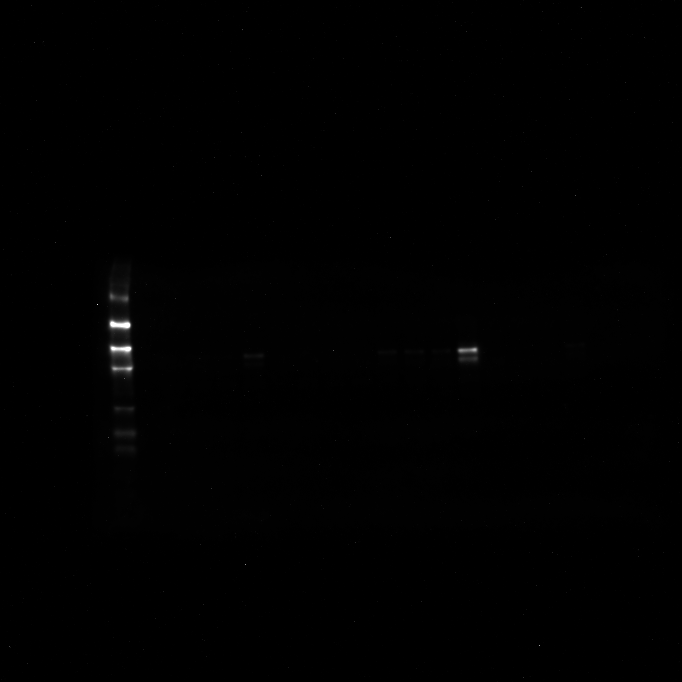

Supplement: Figure 3—source data 1. [file elife-83561-fig3-data1.zip › Figure_3-source_data_1_Figure_3C_p-STAT3.tif]

# Figure 3-source data 1

Figure 3C

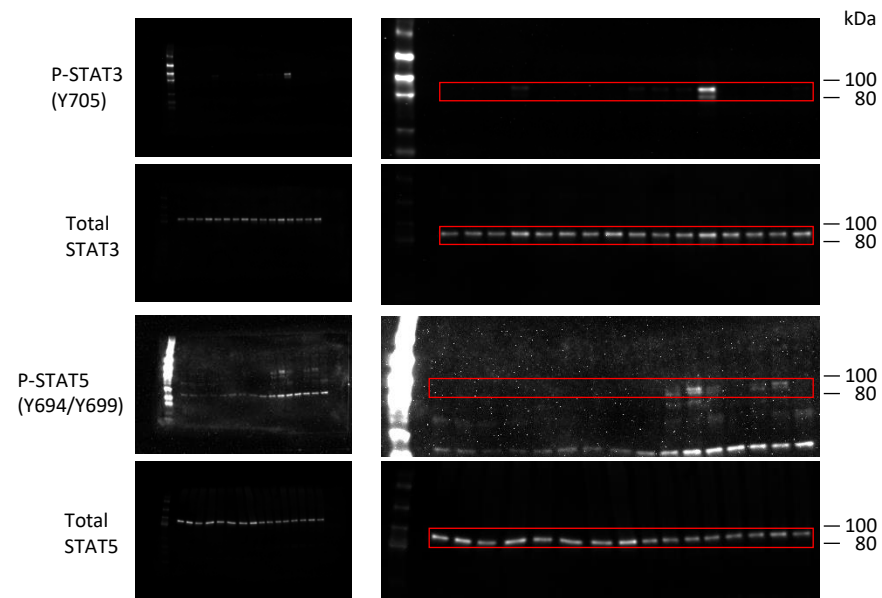

Figure 3D

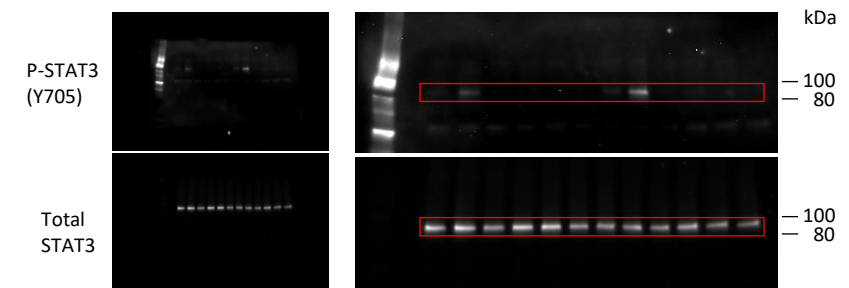

Figure 3I

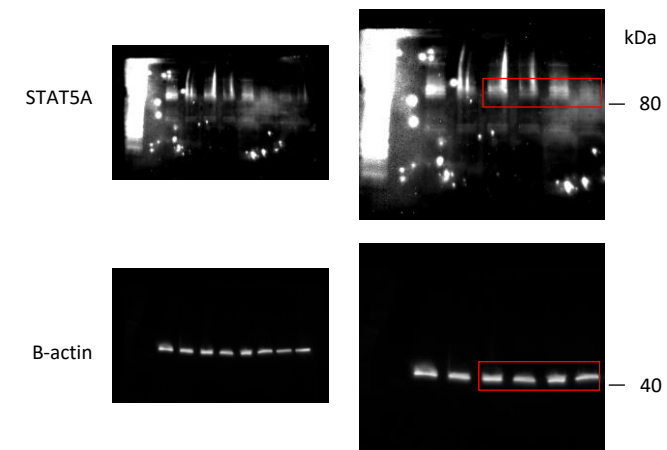

Supplement: Figure 3—source data 2. [file elife-83561-fig3-data2.pdf]

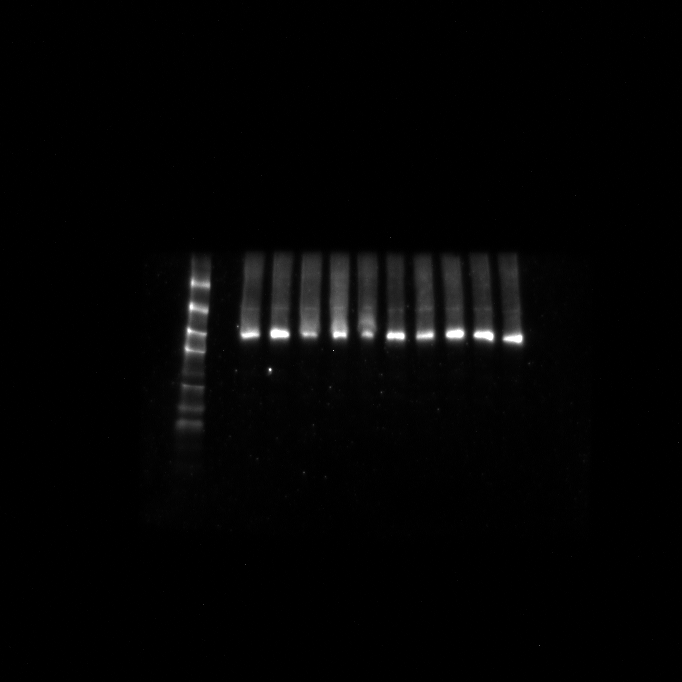

Supplement: Figure 3—figure supplement 1—source data 1. [file elife-83561-fig3-figsupp1-data1.zip › Figure_3-figure_supplement_1-source_data_1_B_total_STAT5.tif]

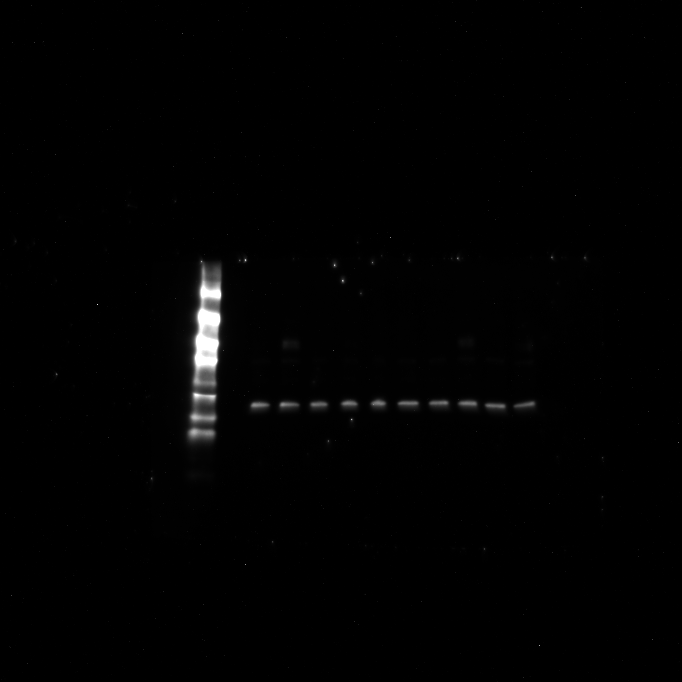

Supplement: Figure 3—figure supplement 1—source data 1. [file elife-83561-fig3-figsupp1-data1.zip › Figure_3-figure_supplement_1-source_data_1_B_p-STAT5.tif]

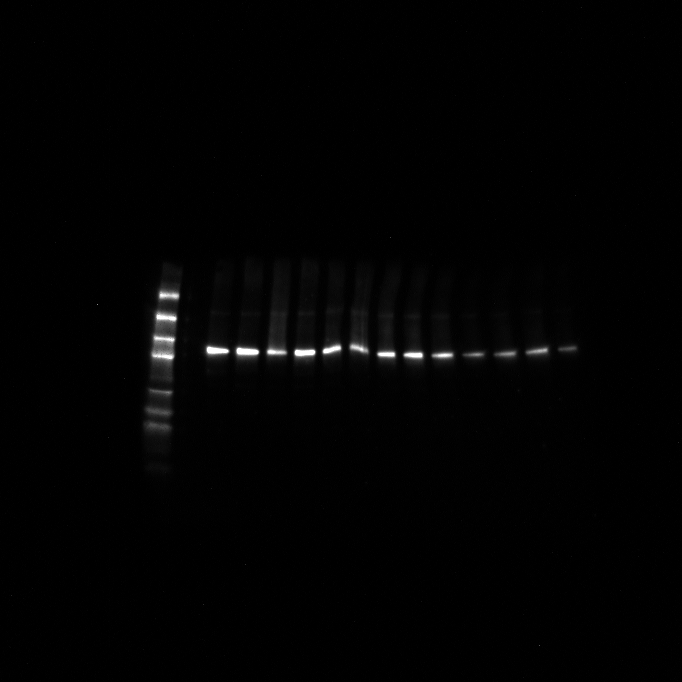

Supplement: Figure 3—figure supplement 1—source data 1. [file elife-83561-fig3-figsupp1-data1.zip › Figure_3-figure_supplement_1-source_data_1_A_total_STAT5.tif]

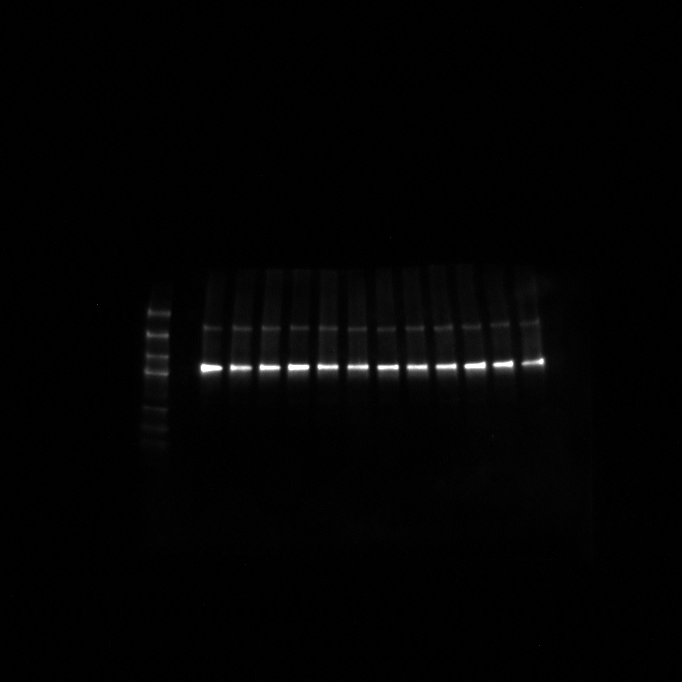

Supplement: Figure 3—figure supplement 1—source data 1. [file elife-83561-fig3-figsupp1-data1.zip › Figure_3-figure_supplement_1-source_data_1_A_total_STAT3.tif]

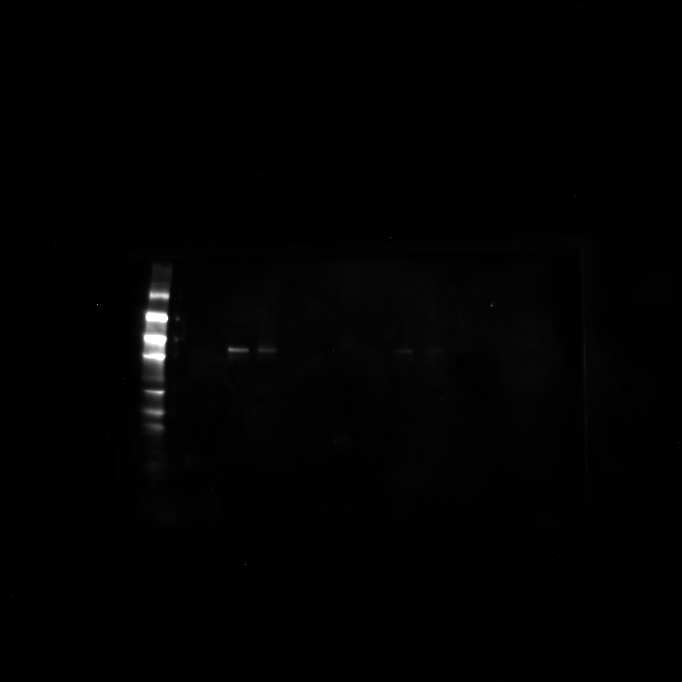

Supplement: Figure 3—figure supplement 1—source data 1. [file elife-83561-fig3-figsupp1-data1.zip › Figure_3-figure_supplement_1-source_data_1_A_p-STAT5.tif]

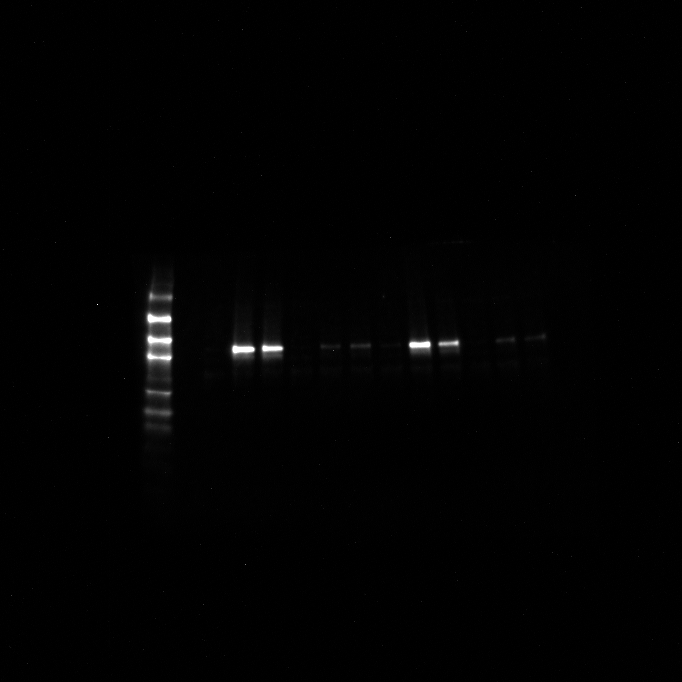

Supplement: Figure 3—figure supplement 1—source data 1. [file elife-83561-fig3-figsupp1-data1.zip › Figure_3-figure_supplement_1-source_data_1_A_p-STAT3.tif]

**Figure 3-figure supplement 1-source data 1**

**A**

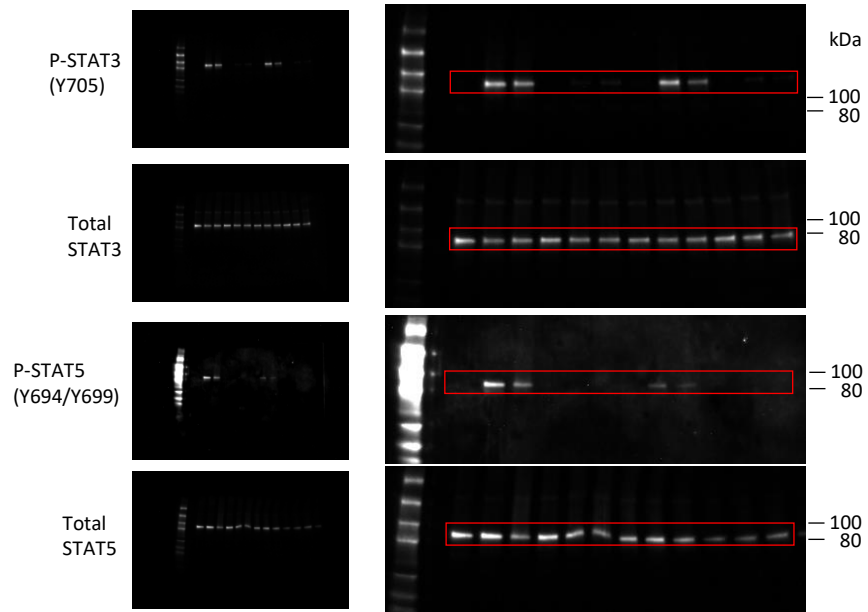

**B**

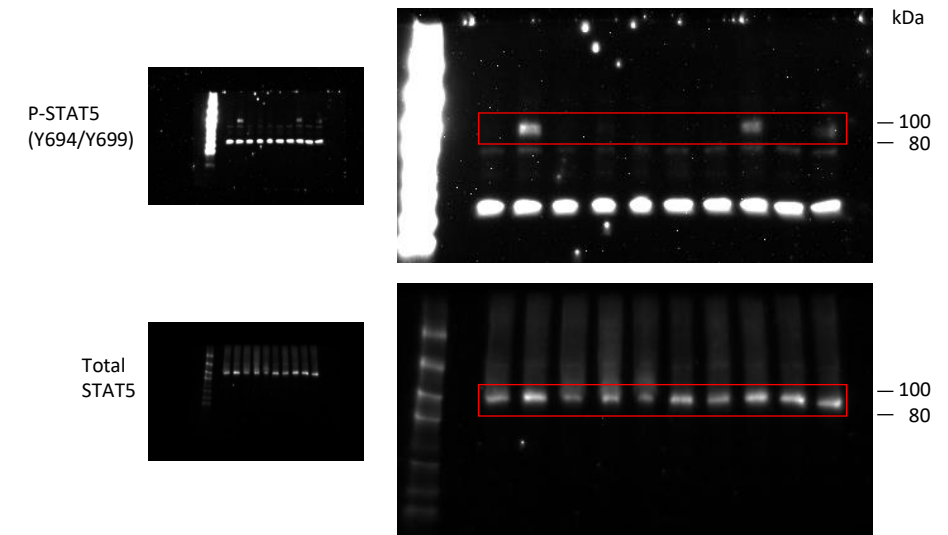

Supplement: Figure 3—figure supplement 1—source data 2. [file elife-83561-fig3-figsupp1-data2.pdf]

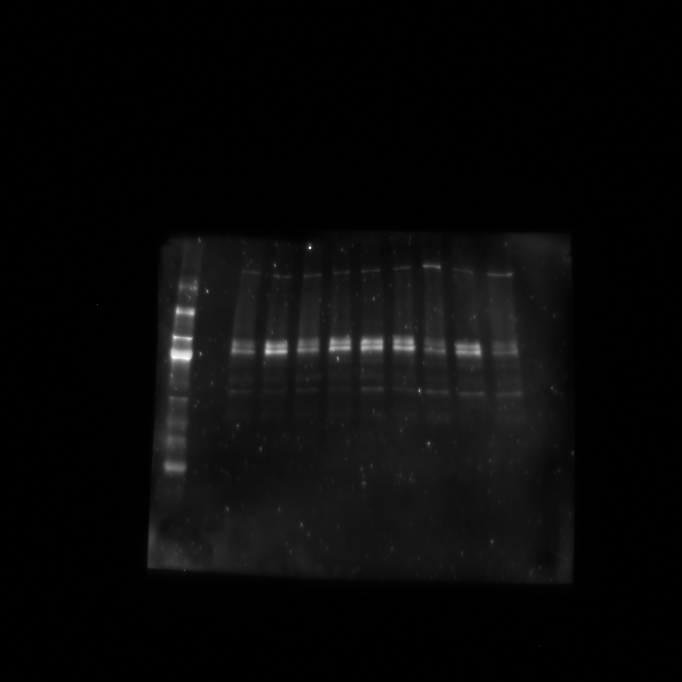

Supplement: Figure 4—source data 1. [file elife-83561-fig4-data1.zip › Figure_4-source_data_1_Figure_4C_total_STAT5.tif]

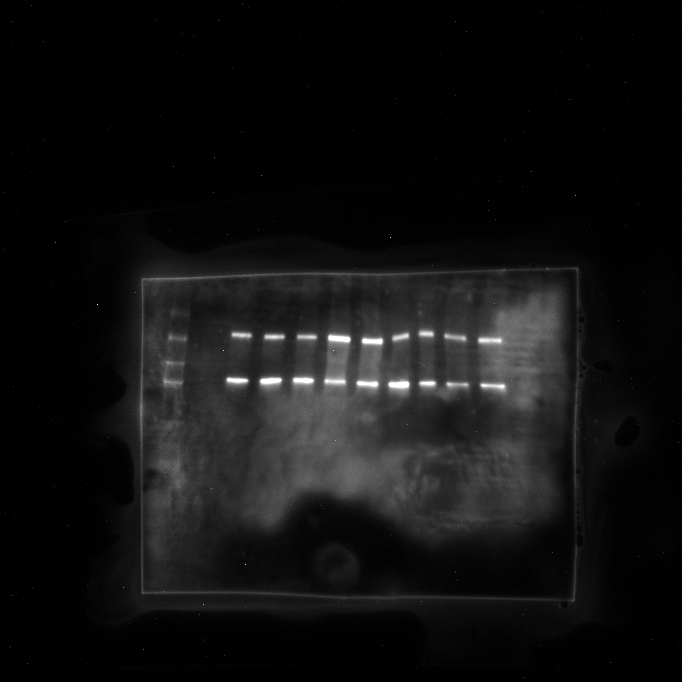

Supplement: Figure 4—source data 1. [file elife-83561-fig4-data1.zip › Figure_4-source_data_1_Figure_4C_total_STAT3.tif]

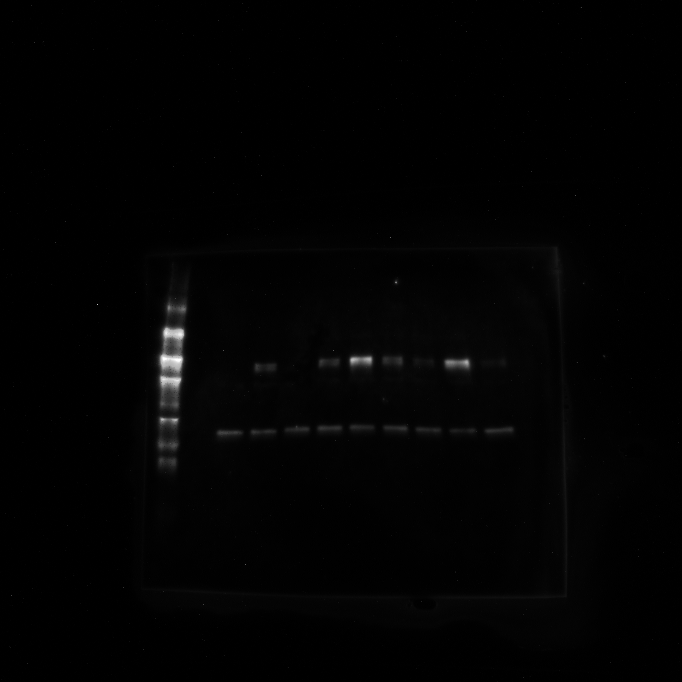

Supplement: Figure 4—source data 1. [file elife-83561-fig4-data1.zip › Figure_4-source_data_1_Figure_4C_p-STAT5.tif]

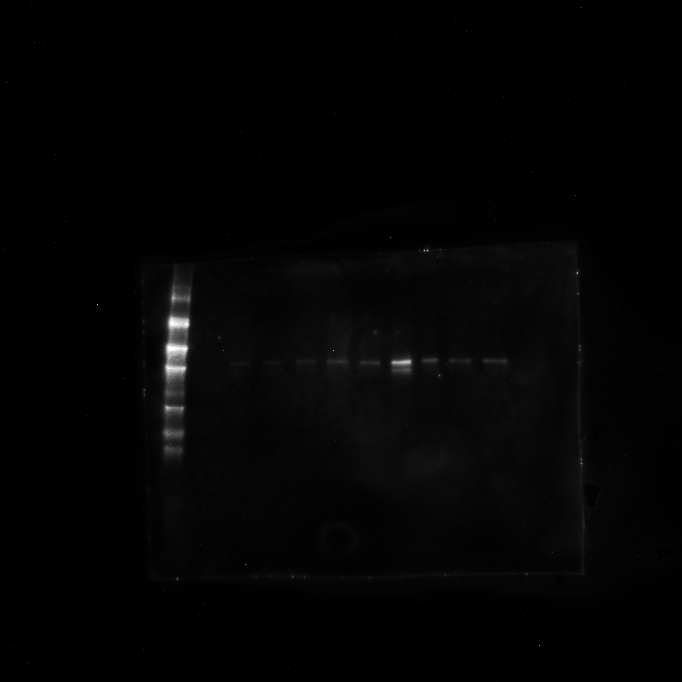

Supplement: Figure 4—source data 1. [file elife-83561-fig4-data1.zip › Figure_4-source_data_1_Figure_4C_p-STAT3.tif]

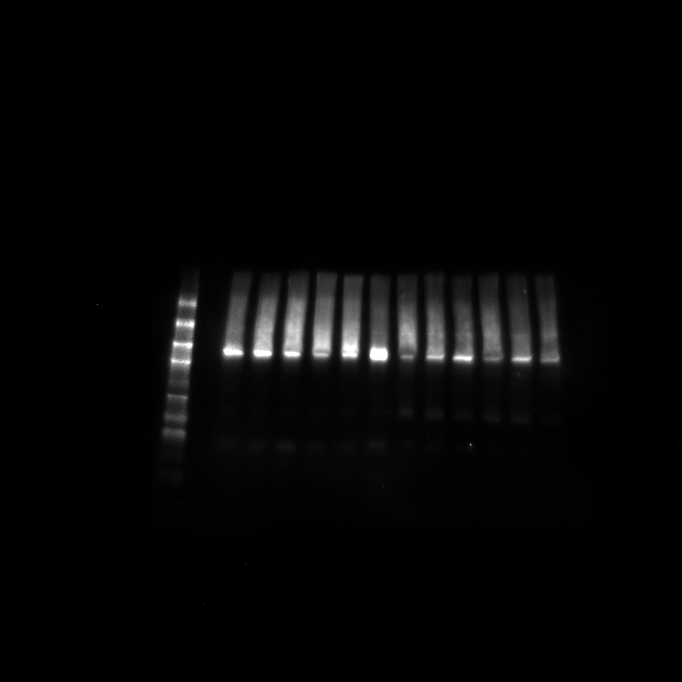

Supplement: Figure 4—source data 1. [file elife-83561-fig4-data1.zip › Figure_4-source_data_1_Figure_4A_total_STAT5A.tif]

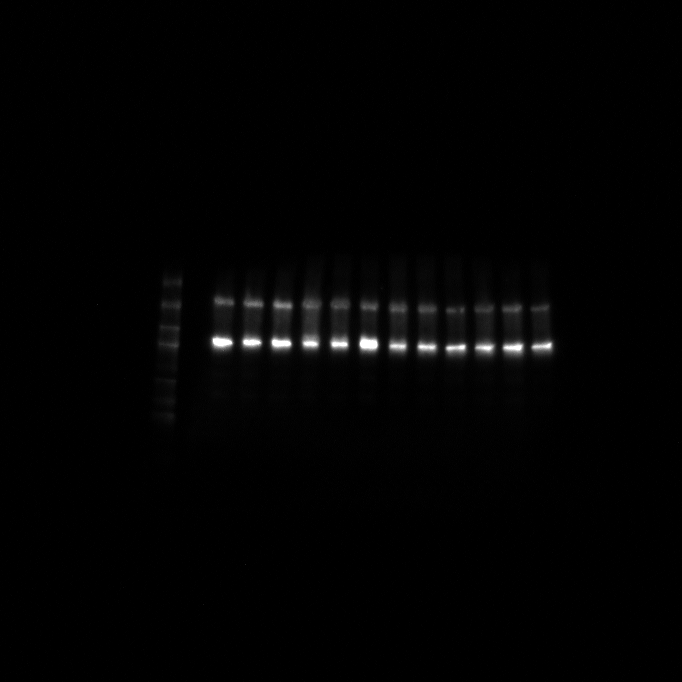

Supplement: Figure 4—source data 1. [file elife-83561-fig4-data1.zip › Figure_4-source_data_1_Figure_4A_total_STAT3.tif]

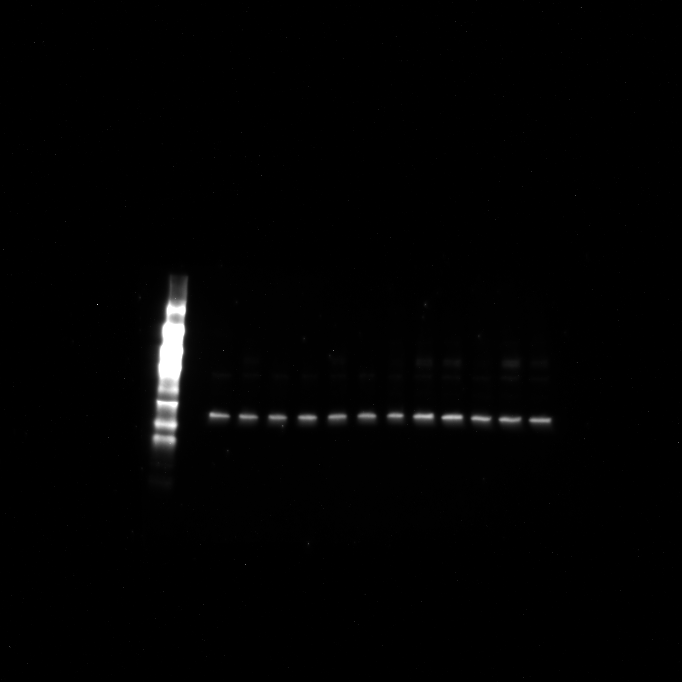

Supplement: Figure 4—source data 1. [file elife-83561-fig4-data1.zip › Figure_4-source_data_1_Figure_4A_p-STAT5A.tif]

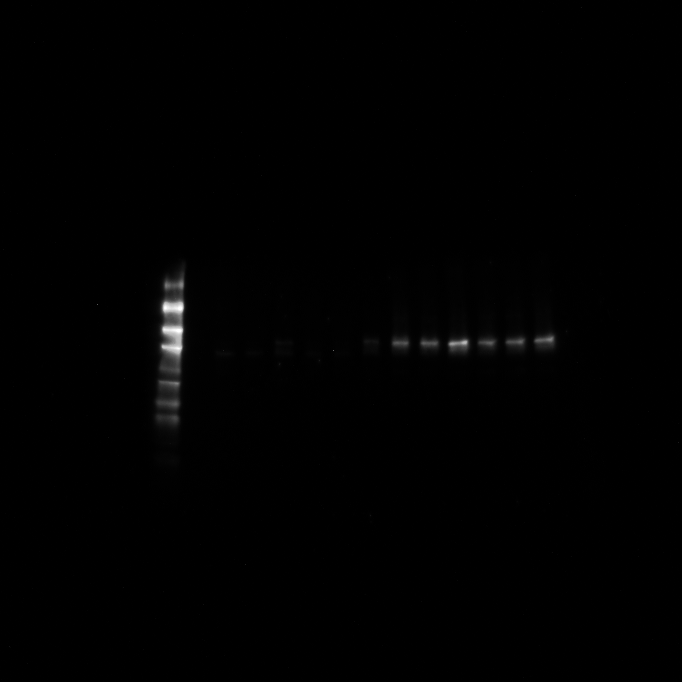

Supplement: Figure 4—source data 1. [file elife-83561-fig4-data1.zip › Figure_4-source_data_1_Figure_4A_p-STAT3.tif]

# Figure 4-source data 1

Figure 4A

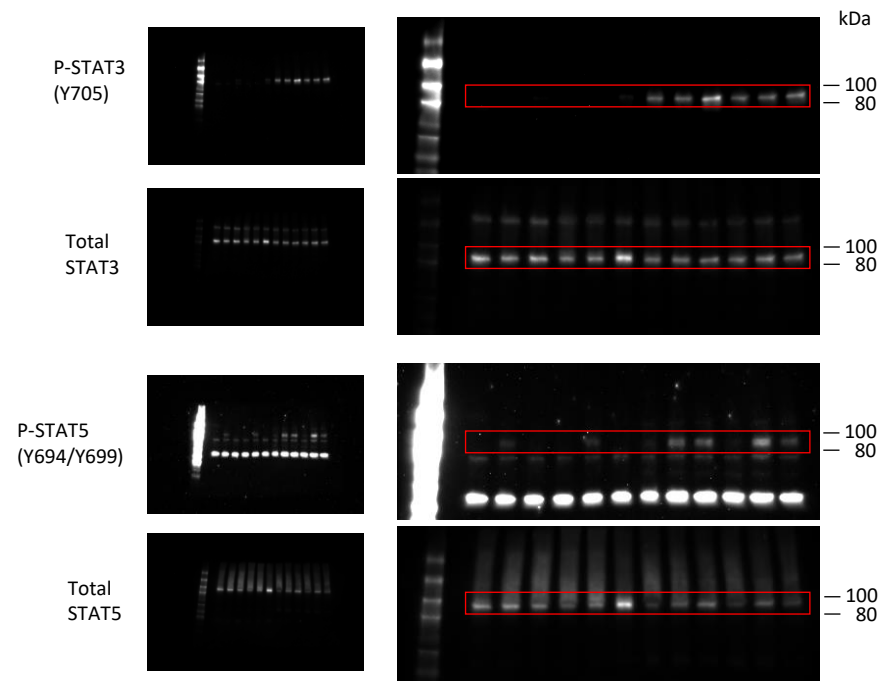

Figure 4C

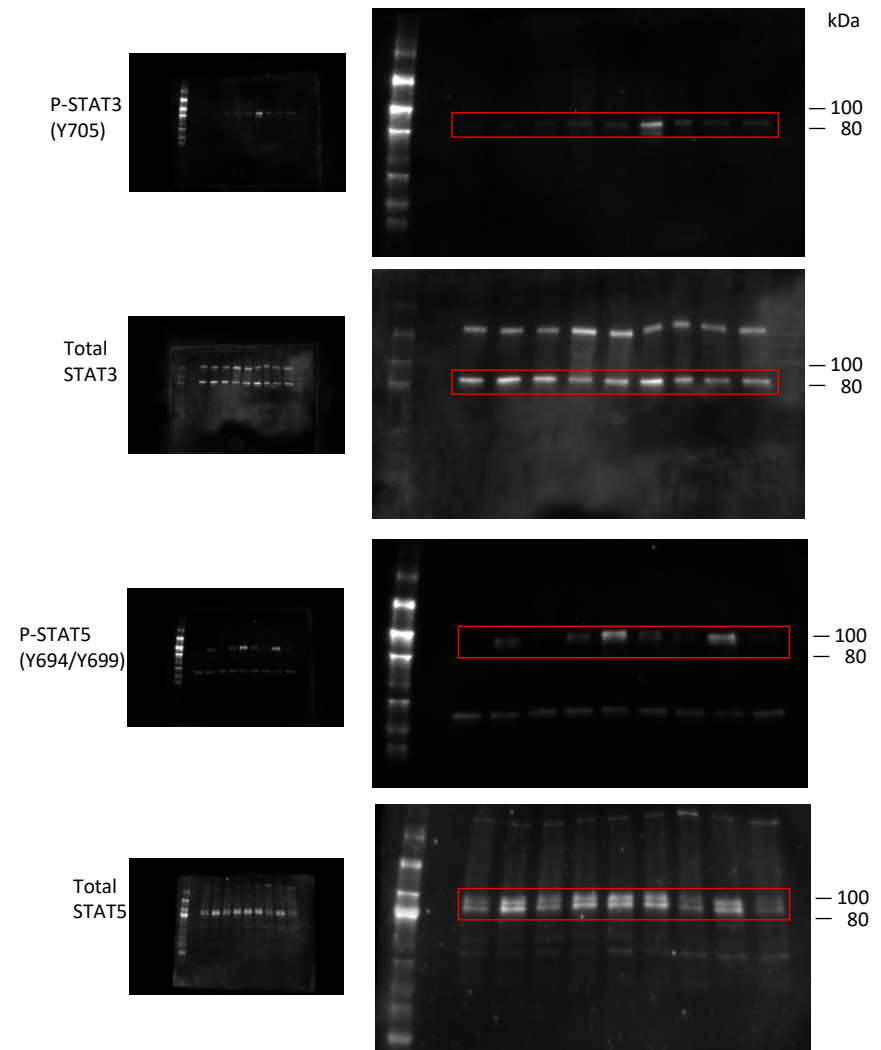

Supplement: Figure 4—source data 2. [file elife-83561-fig4-data2.pdf]

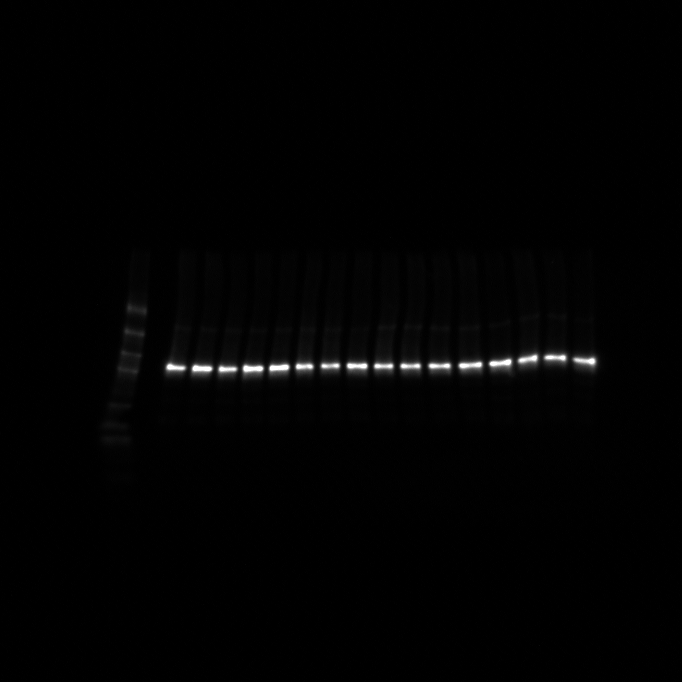

Supplement: Figure 4—figure supplement 1—source data 1. [file elife-83561-fig4-figsupp1-data1.zip › Figure_4-figure_supplement_1-source_data_1_total_STAT3.tif]

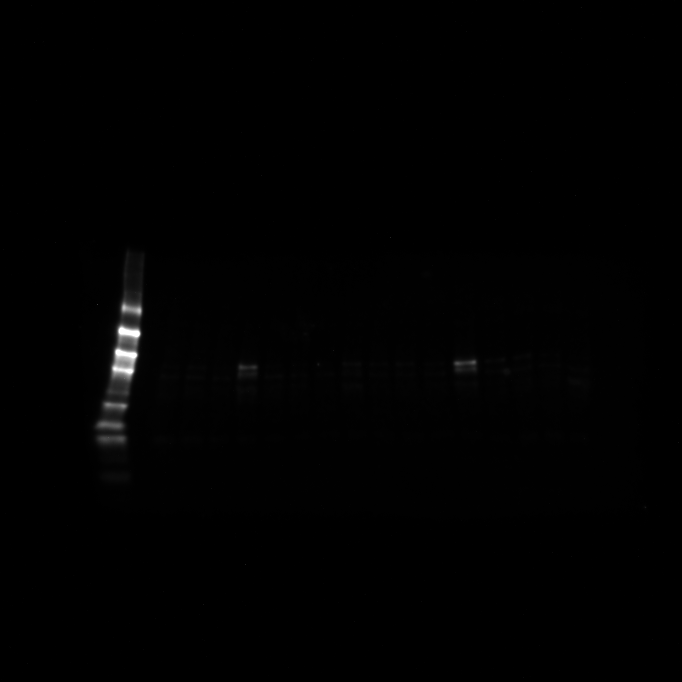

Supplement: Figure 4—figure supplement 1—source data 1. [file elife-83561-fig4-figsupp1-data1.zip › Figure_4-figure_supplement_1-source_data_1_p-STAT3.tif]

**Figure 4-figure supplement 1-source data 1**

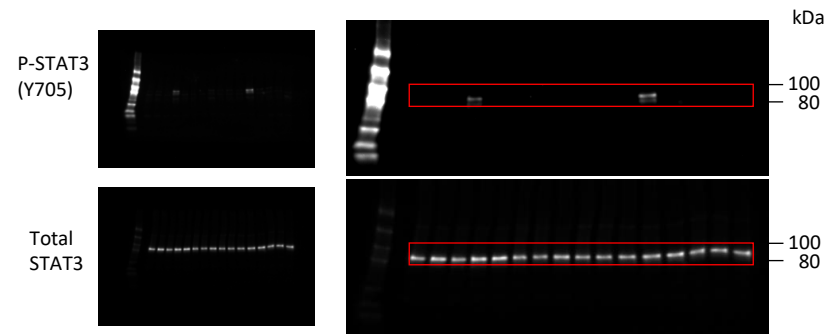

Supplement: Figure 4—figure supplement 1—source data 2. [file elife-83561-fig4-figsupp1-data2.pdf]

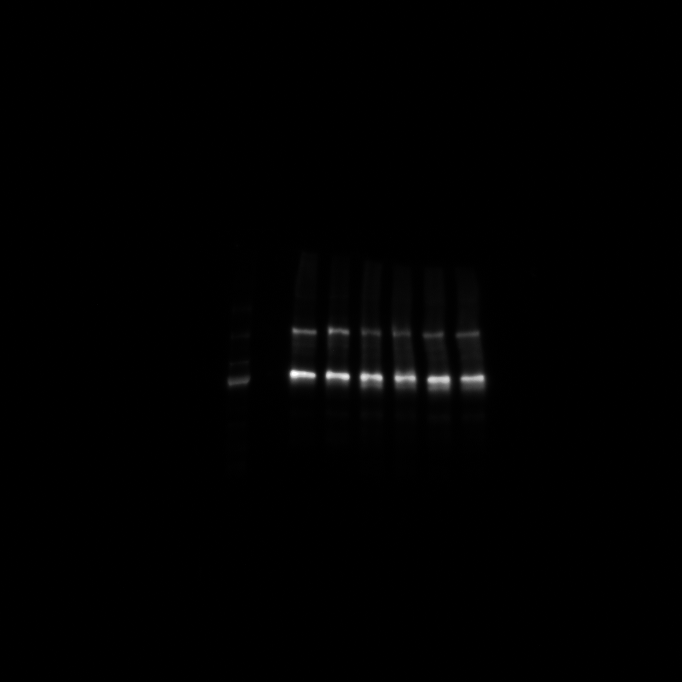

Supplement: Figure 4—figure supplement 2—source data 1. [file elife-83561-fig4-figsupp2-data1.zip › Figure_4-figure_supplement_2-source_data_1_C_total_STAT3.tif]

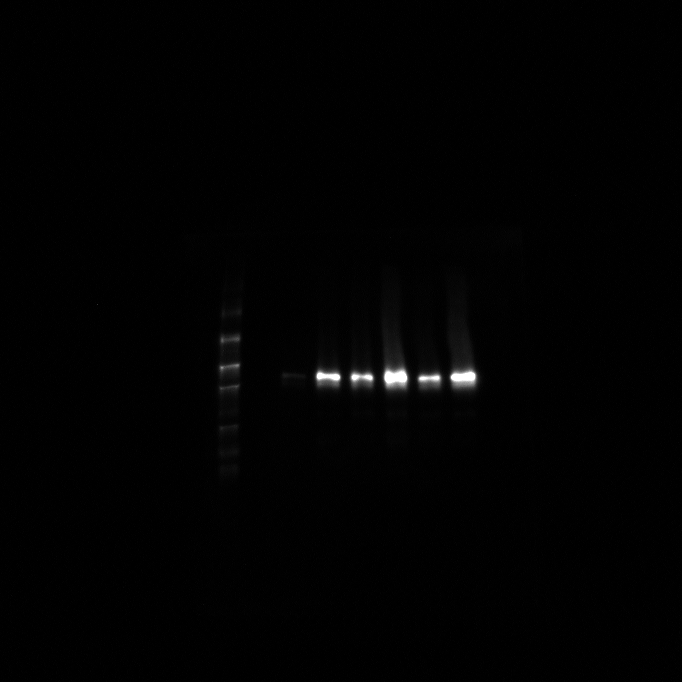

Supplement: Figure 4—figure supplement 2—source data 1. [file elife-83561-fig4-figsupp2-data1.zip › Figure_4-figure_supplement_2-source_data_1_C_p-STAT3.tif]

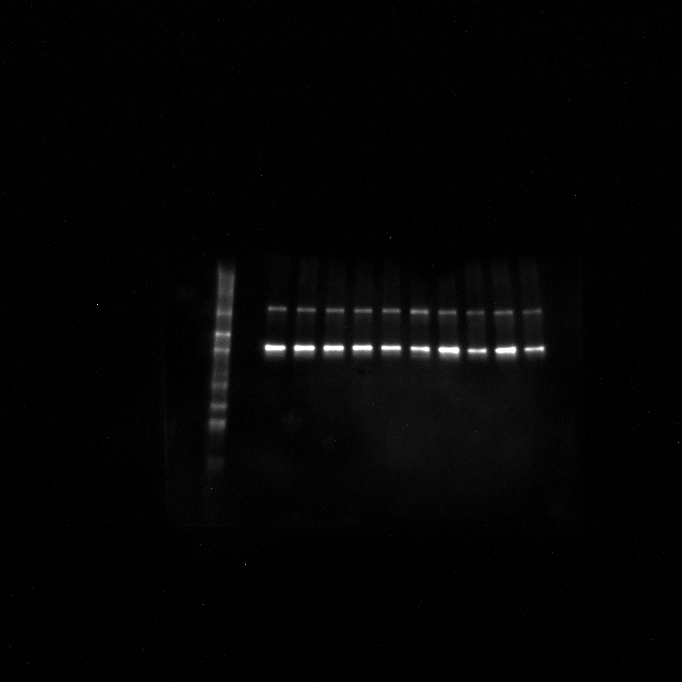

Supplement: Figure 4—figure supplement 2—source data 1. [file elife-83561-fig4-figsupp2-data1.zip › Figure_4-figure_supplement_2-source_data_1_B_total_STAT3.tif]

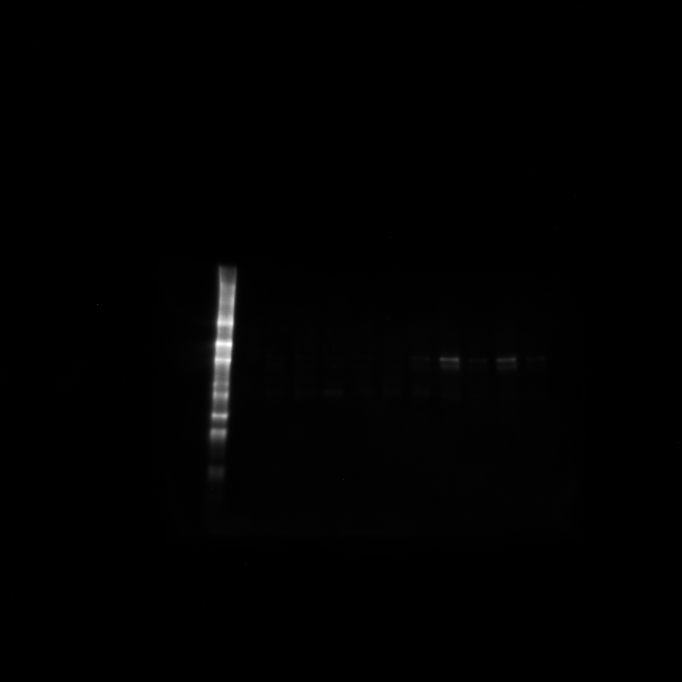

Supplement: Figure 4—figure supplement 2—source data 1. [file elife-83561-fig4-figsupp2-data1.zip › Figure_4-figure_supplement_2-source_data_1_B_p-STAT3.tif]

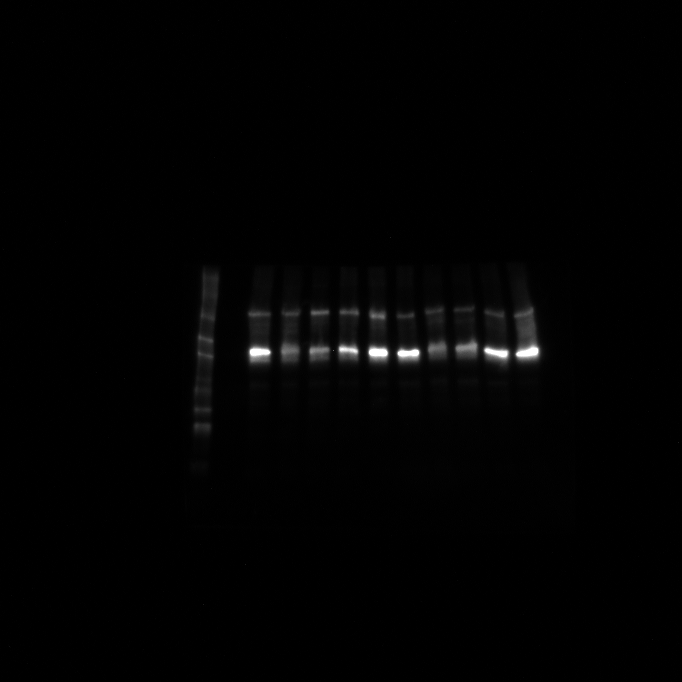

Supplement: Figure 4—figure supplement 2—source data 1. [file elife-83561-fig4-figsupp2-data1.zip › Figure_4-figure_supplement_2-source_data_1_A_total_STAT3.tif]

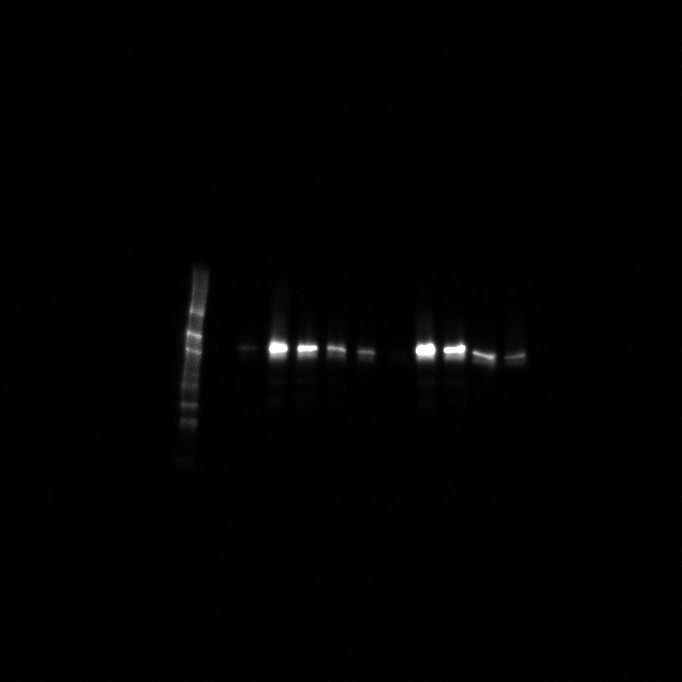

Supplement: Figure 4—figure supplement 2—source data 1. [file elife-83561-fig4-figsupp2-data1.zip › Figure_4-figure_supplement_2-source_data_1_A_p-STAT3.tif]

**Figure 4-figure supplement 2-source data 1**

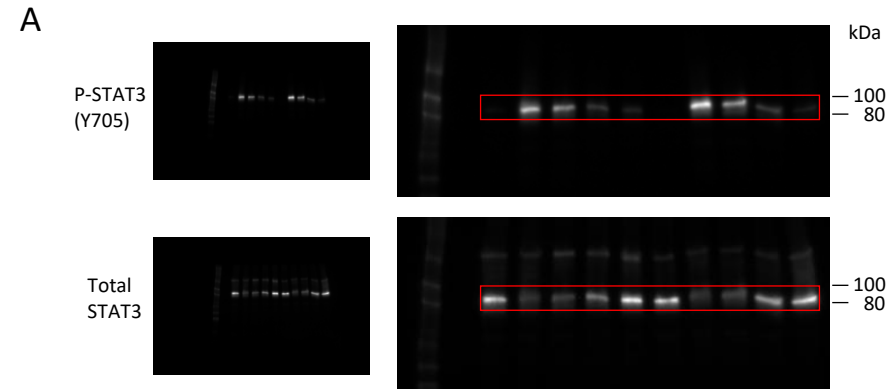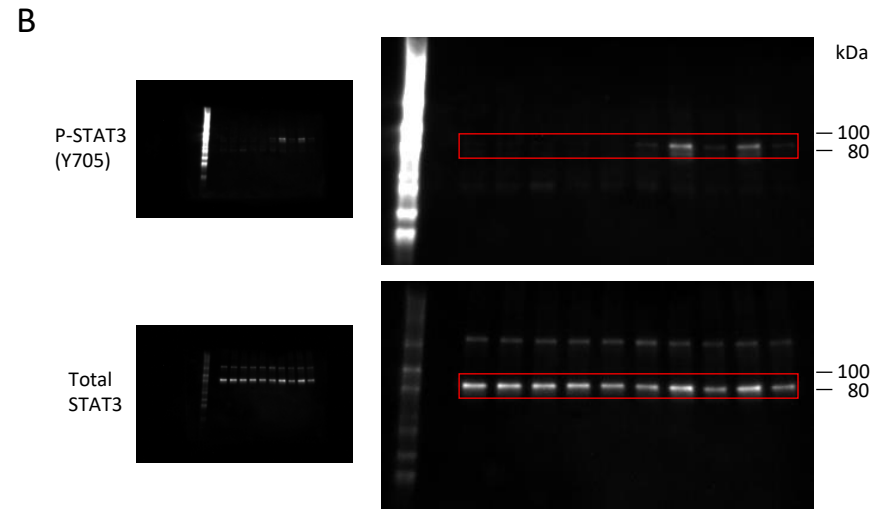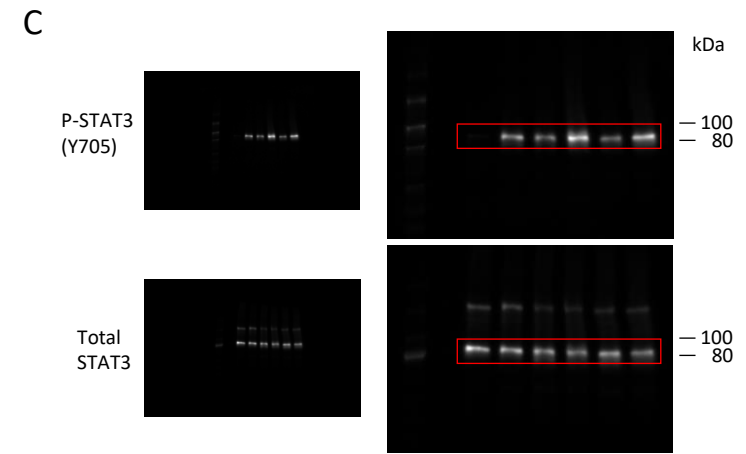

Supplement: Figure 4—figure supplement 2—source data 2. [file elife-83561-fig4-figsupp2-data2.pdf]

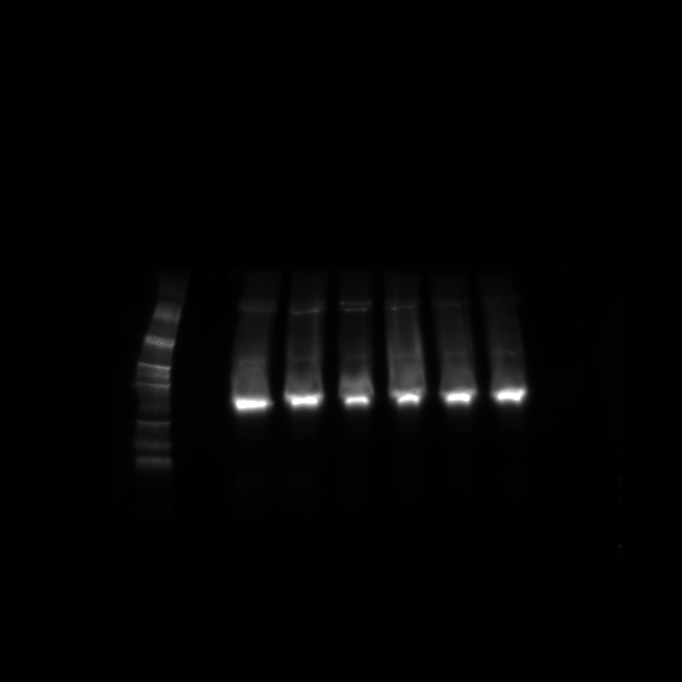

Supplement: Figure 4—figure supplement 3—source data 1. [file elife-83561-fig4-figsupp3-data1.zip › Figure_4-figure_supplement_3-source_data_1_total_p65.tif]

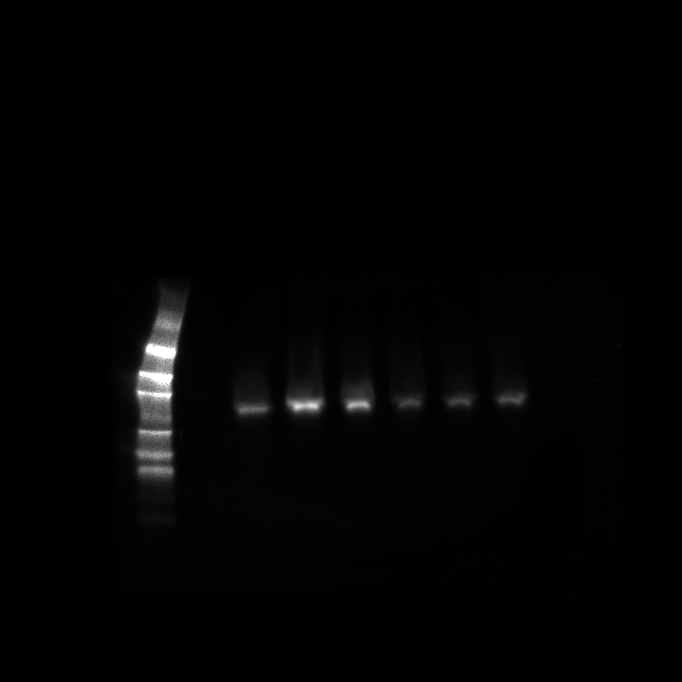

Supplement: Figure 4—figure supplement 3—source data 1. [file elife-83561-fig4-figsupp3-data1.zip › Figure_4-figure_supplement_3-source_data_1_p-p65.tif]

**Figure 4-figure supplement 3-source data 1**

D

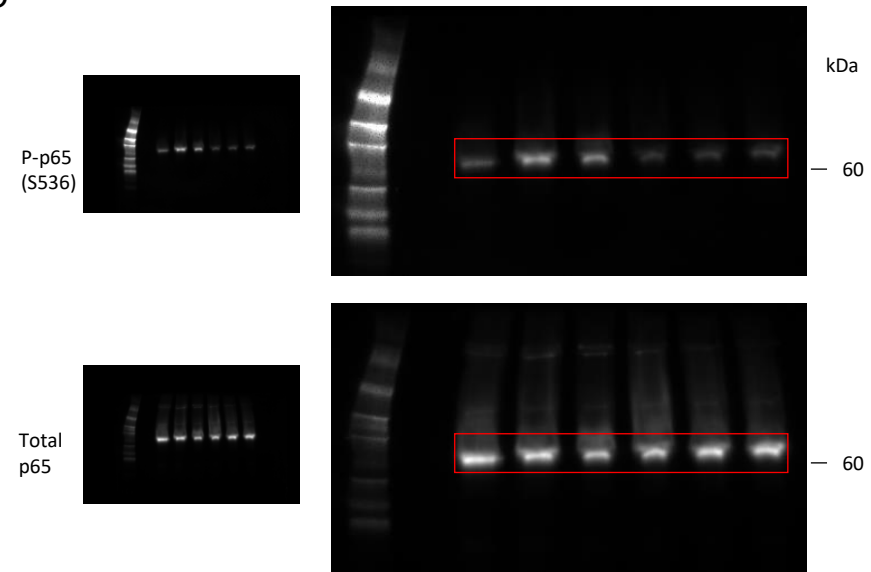

Supplement: Figure 4—figure supplement 3—source data 2. [file elife-83561-fig4-figsupp3-data2.pdf]

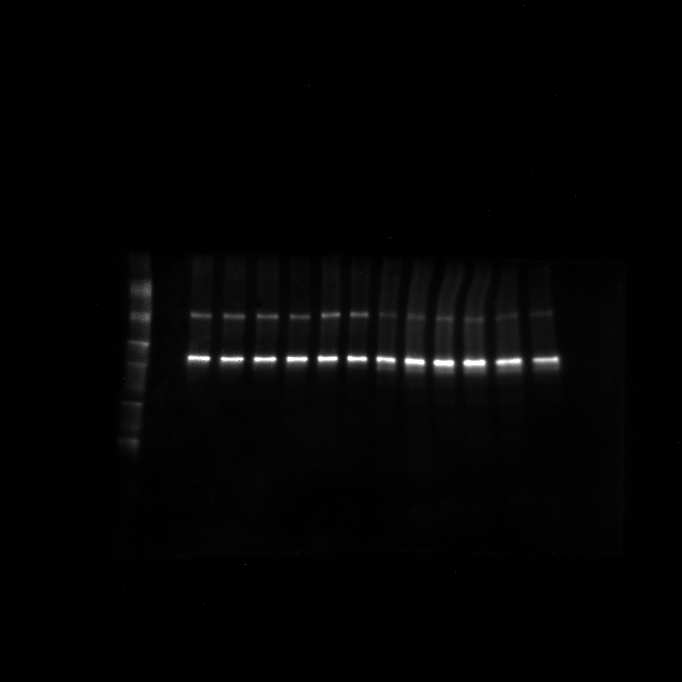

Supplement: Figure 5—source data 1. [file elife-83561-fig5-data1.zip › Figure_5-source_data_1_Figure_5B_total_STAT3.tif]

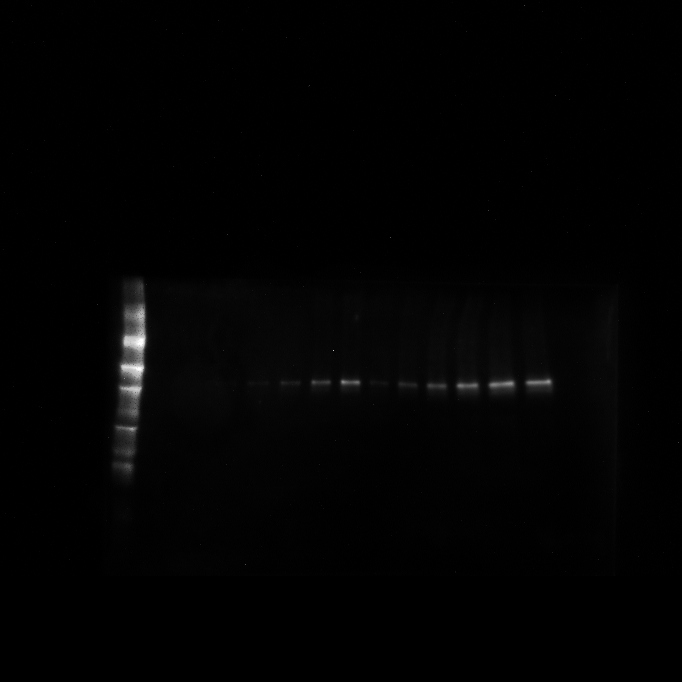

Supplement: Figure 5—source data 1. [file elife-83561-fig5-data1.zip › Figure_5-source_data_1_Figure_5B_p-STAT3.tif]

# Figure 5-source data 1

Figure 5B

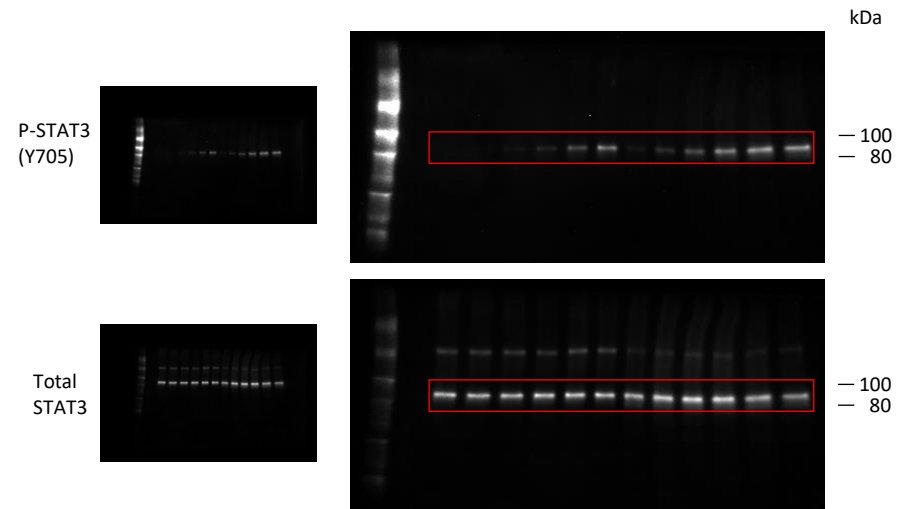

Supplement: Figure 5—source data 2. [file elife-83561-fig5-data2.pdf]

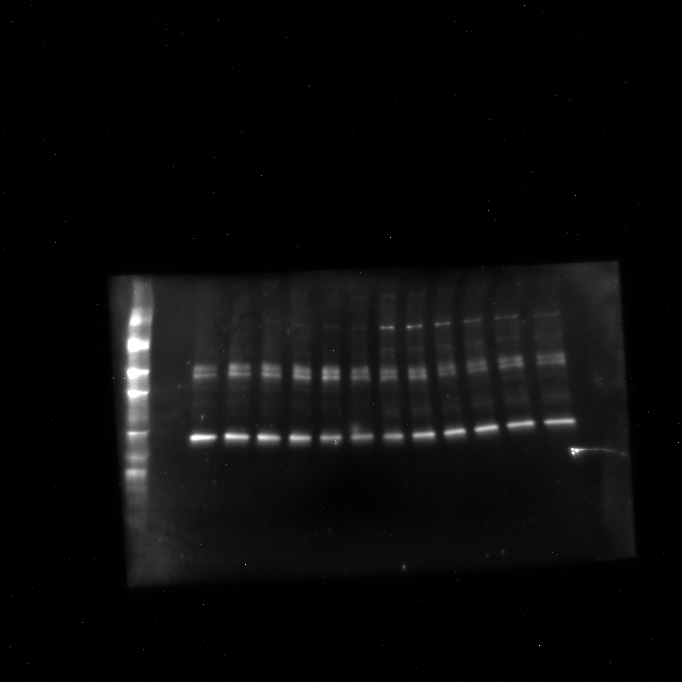

Supplement: Figure 6—source data 1. [file elife-83561-fig6-data1.zip › Figure_6-source_data_1_Figure_6D_total_STAT5.tif]

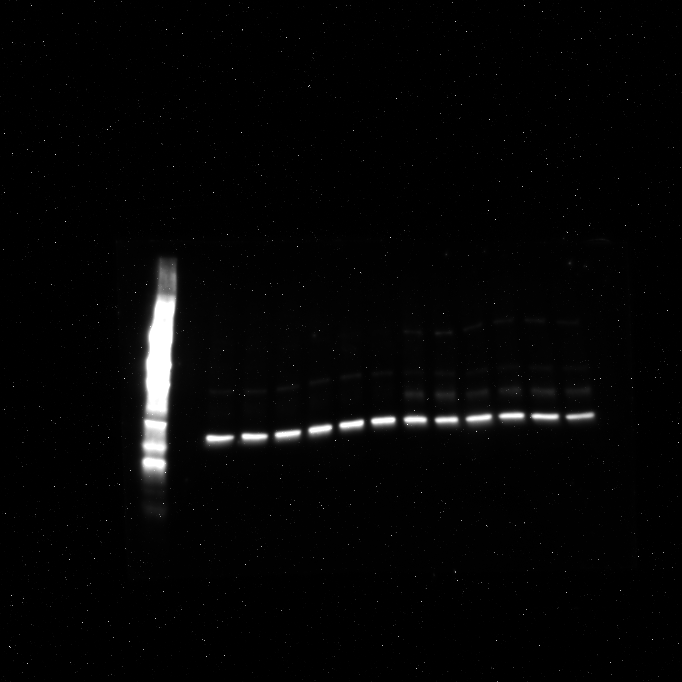

Supplement: Figure 6—source data 1. [file elife-83561-fig6-data1.zip › Figure_6-source_data_1_Figure_6D_p-STAT5.tif]

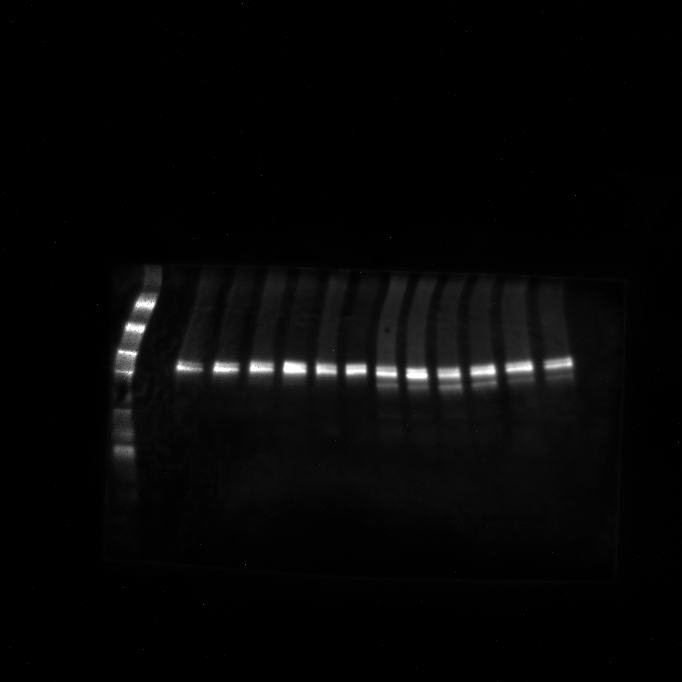

Supplement: Figure 6—source data 1. [file elife-83561-fig6-data1.zip › Figure_6-source_data_1_Figure_6B_total_STAT5.tif]

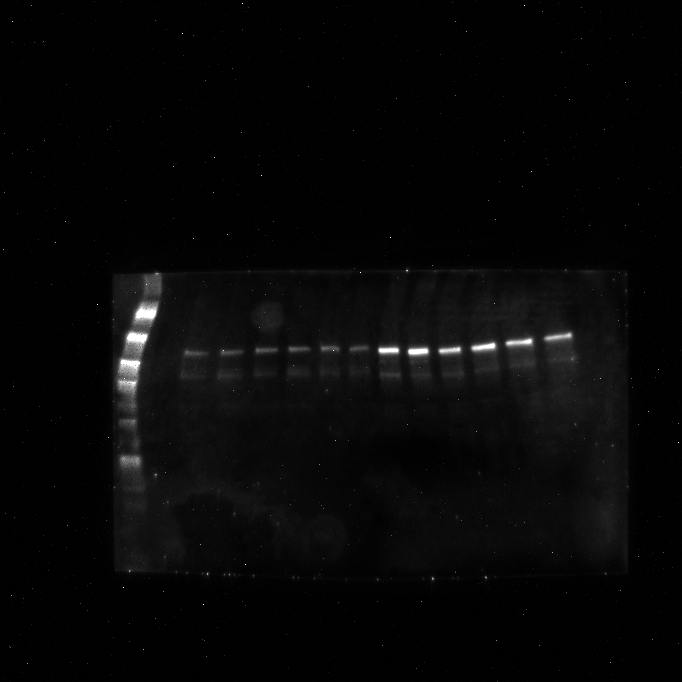

Supplement: Figure 6—source data 1. [file elife-83561-fig6-data1.zip › Figure_6-source_data_1_Figure_6B_total_FAK.tif]

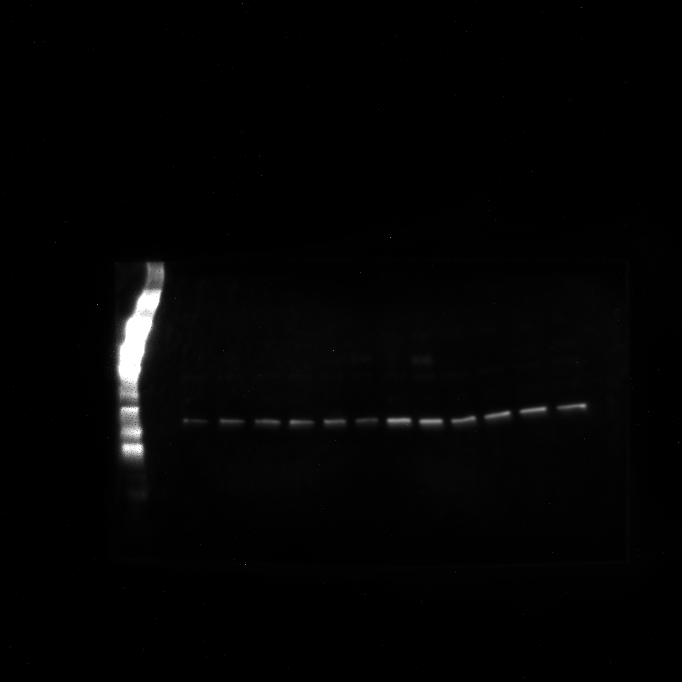

Supplement: Figure 6—source data 1. [file elife-83561-fig6-data1.zip › Figure_6-source_data_1_Figure_6B_p-STAT5.tif]

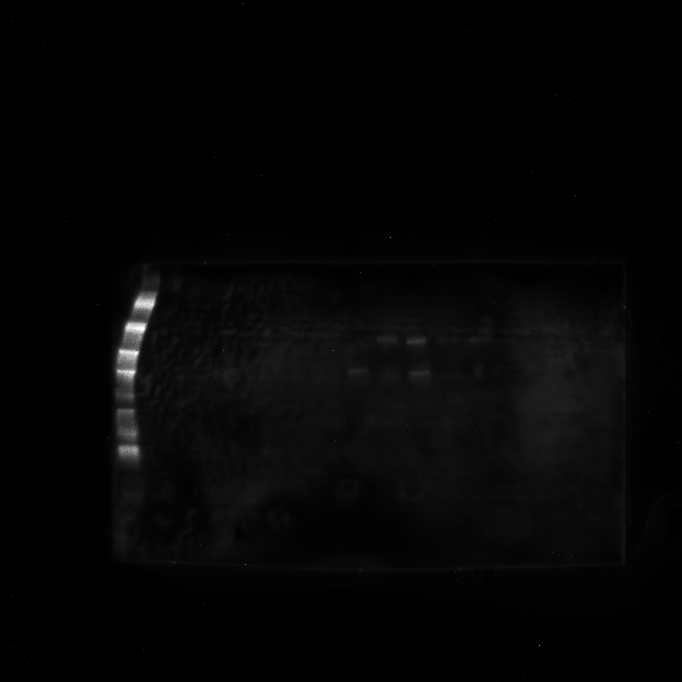

Supplement: Figure 6—source data 1. [file elife-83561-fig6-data1.zip › Figure_6-source_data_1_Figure_6B_p-FAK.tif]

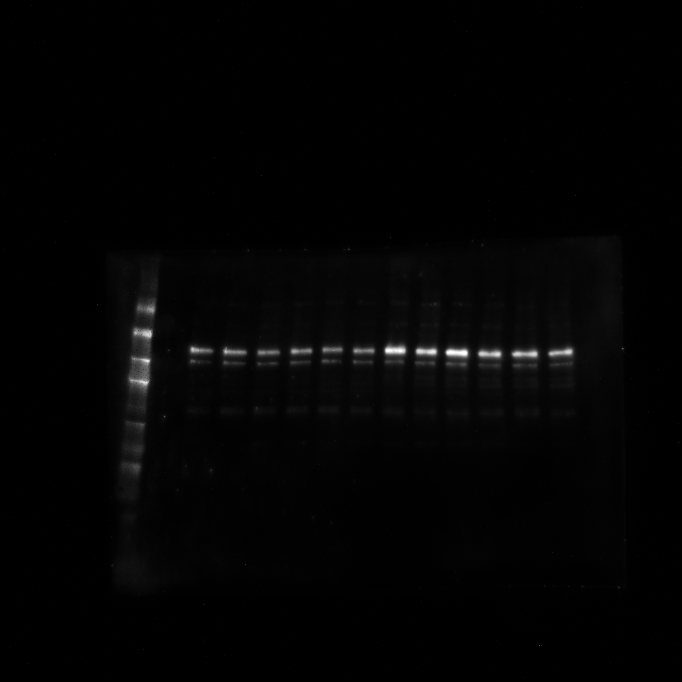

Supplement: Figure 6—source data 1. [file elife-83561-fig6-data1.zip › Figure_6-source_data_1_Figure_6A_total_FAK.tif]

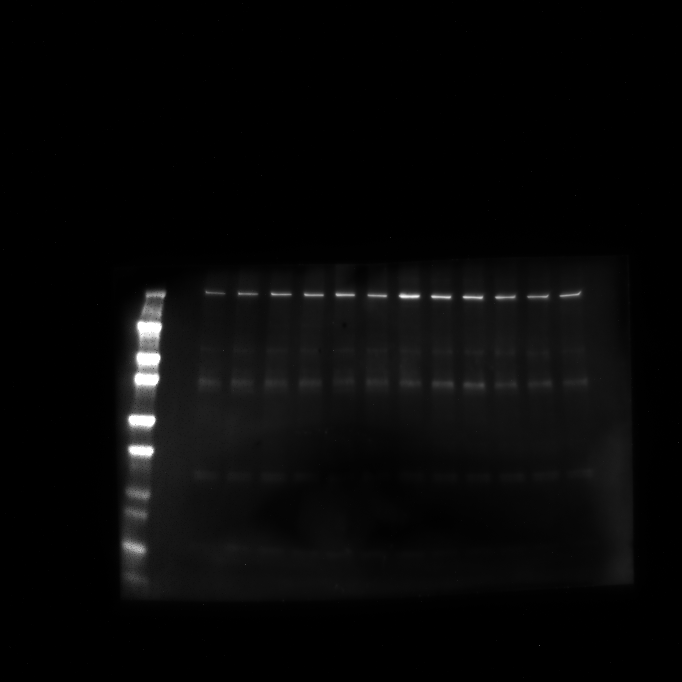

Supplement: Figure 6—source data 1. [file elife-83561-fig6-data1.zip › Figure_6-source_data_1_Figure_6A_pan_PKC.tif]

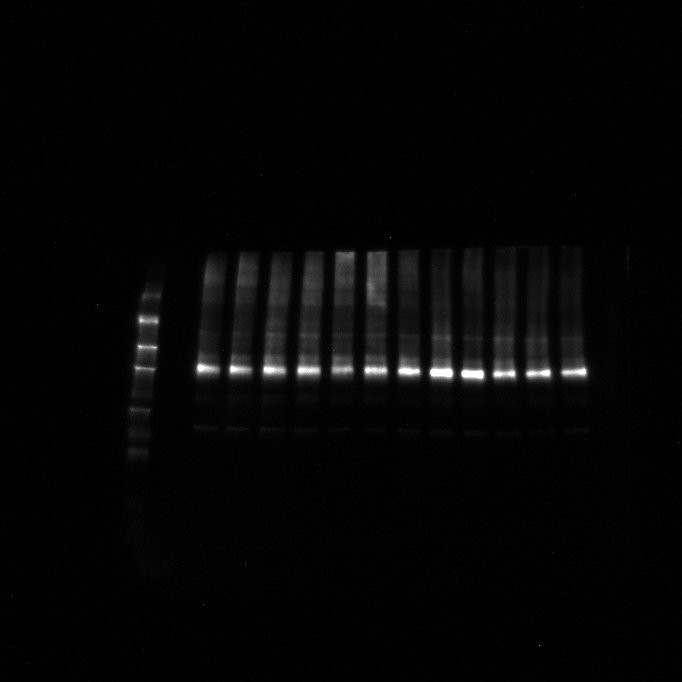

Supplement: Figure 6—source data 1. [file elife-83561-fig6-data1.zip › Figure_6-source_data_1_Figure_6A_p-PKC.tif]

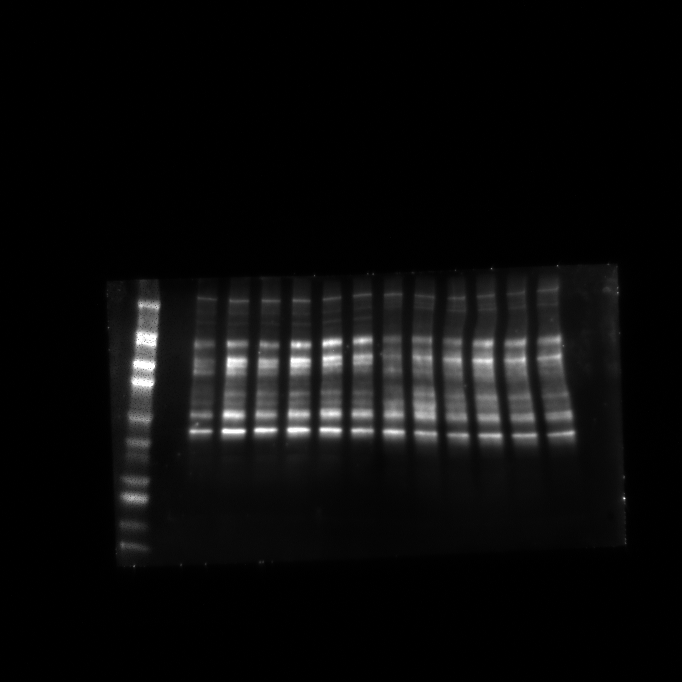

Supplement: Figure 6—source data 1. [file elife-83561-fig6-data1.zip › Figure_6-source_data_1_Figure_6A_p-FAK_Y567.tif]

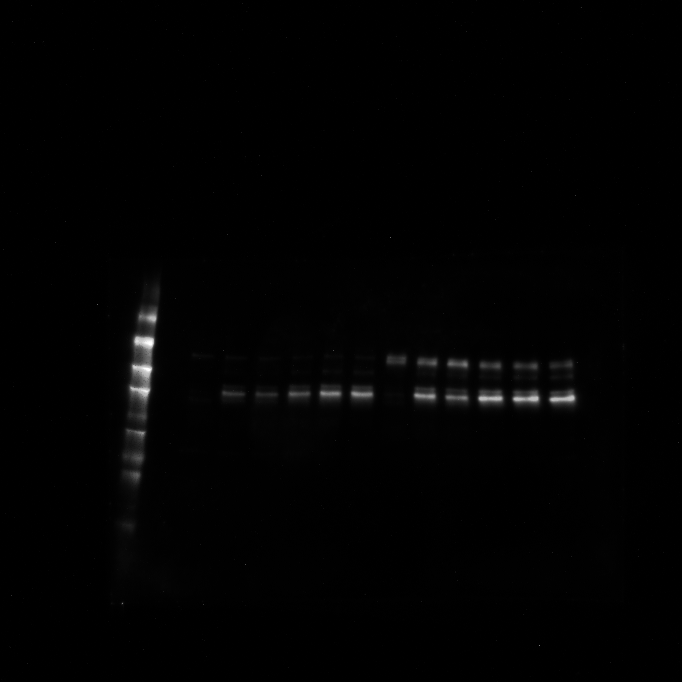

Supplement: Figure 6—source data 1. [file elife-83561-fig6-data1.zip › Figure_6-source_data_1_Figure_6A_p-FAK_Y397.tif]

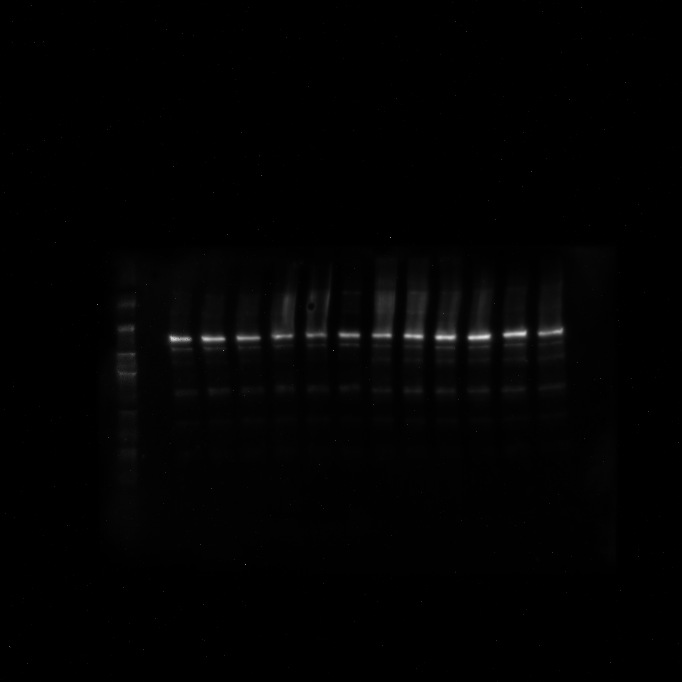

Supplement: Figure 6—source data 1. [file elife-83561-fig6-data1.zip › Figure_6-source_data_1_Figure_6A_b-actin.tif]

Figure 6-source data 1

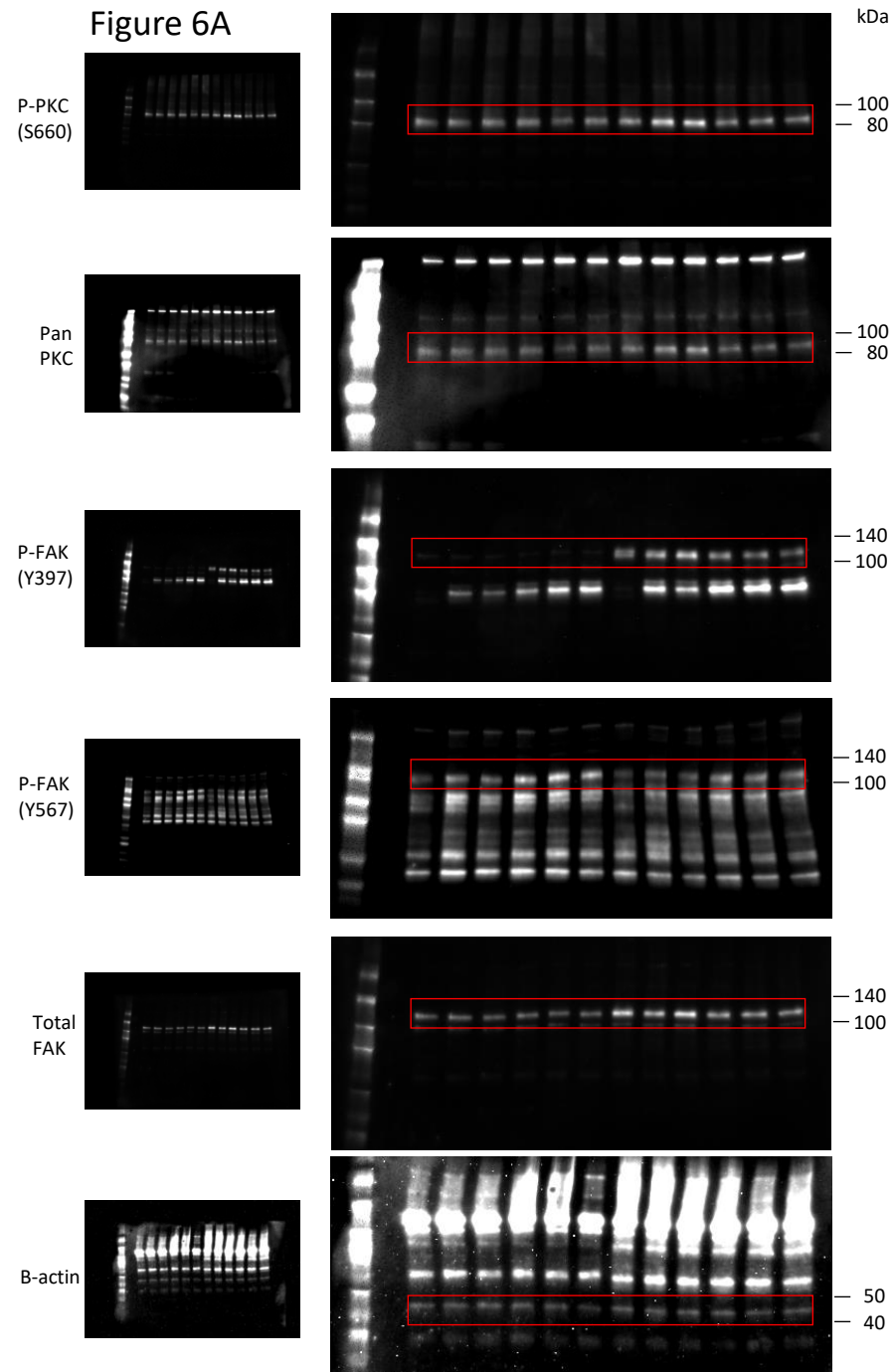

Figure 6B

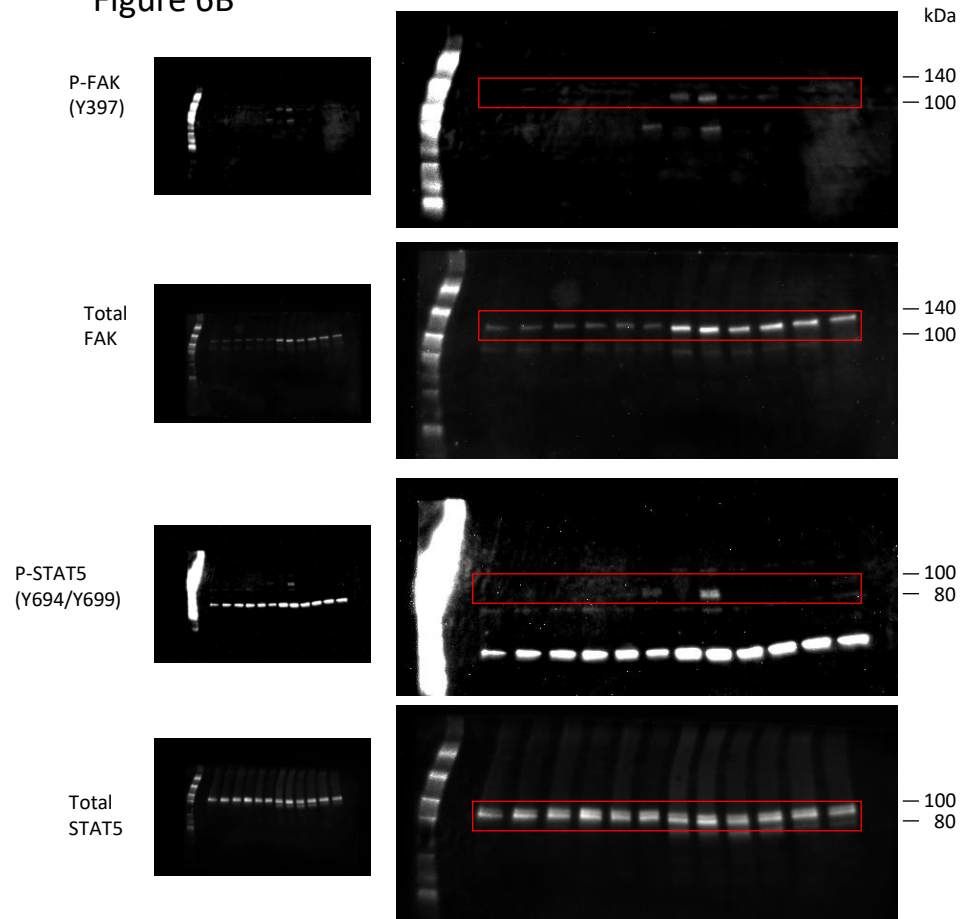

Figure 6D

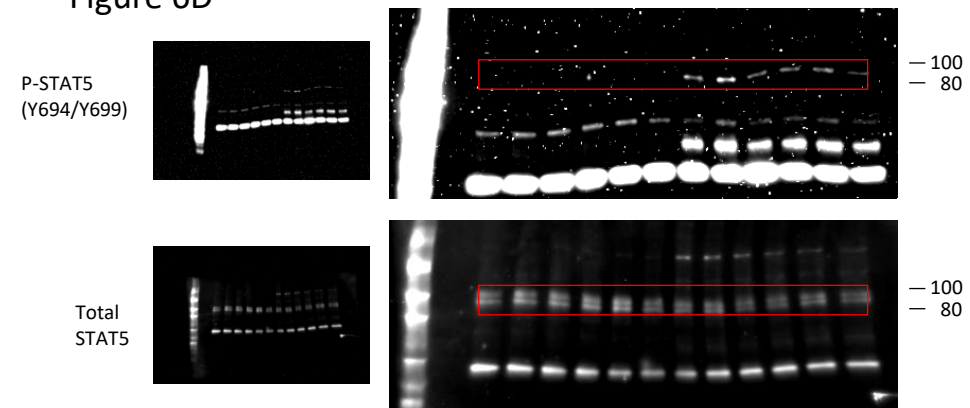

Supplement: Figure 6—source data 2. [file elife-83561-fig6-data2.pdf]

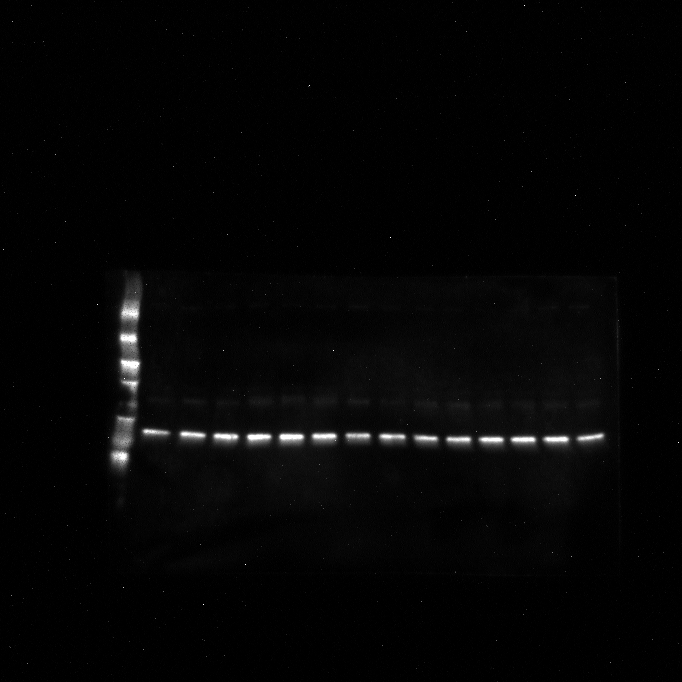

Supplement: Figure 6—figure supplement 2—source data 1. [file elife-83561-fig6-figsupp2-data1.zip › Figure 6-figure supplement 2-source data 1 A b-actin.tif]

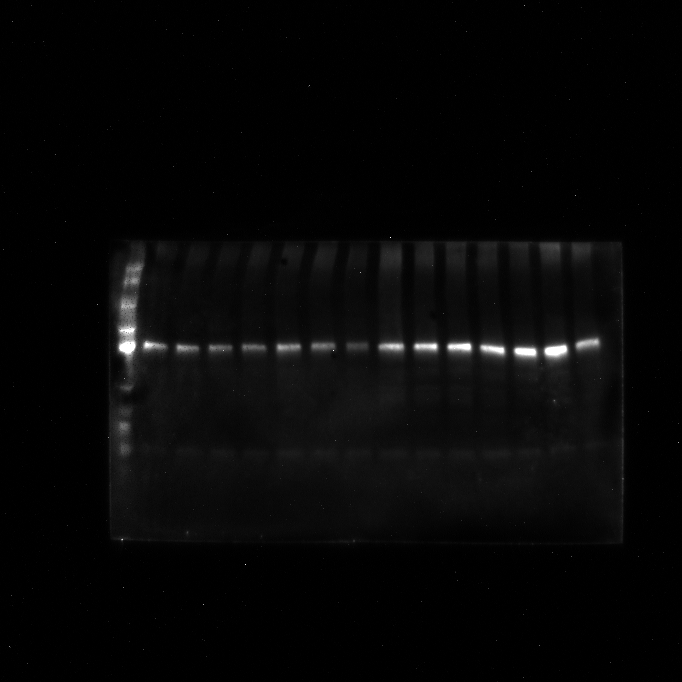

Supplement: Figure 6—figure supplement 2—source data 1. [file elife-83561-fig6-figsupp2-data1.zip › Figure 6-figure supplement 2-source data 1 A pan PKC.tif]

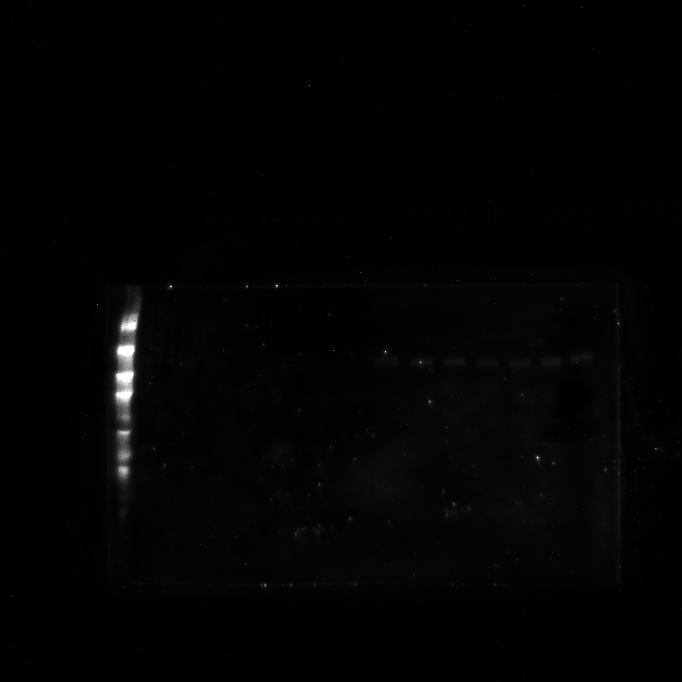

Supplement: Figure 6—figure supplement 2—source data 1. [file elife-83561-fig6-figsupp2-data1.zip › Figure 6-figure supplement 2-source data 1 A p-FAK.tif]

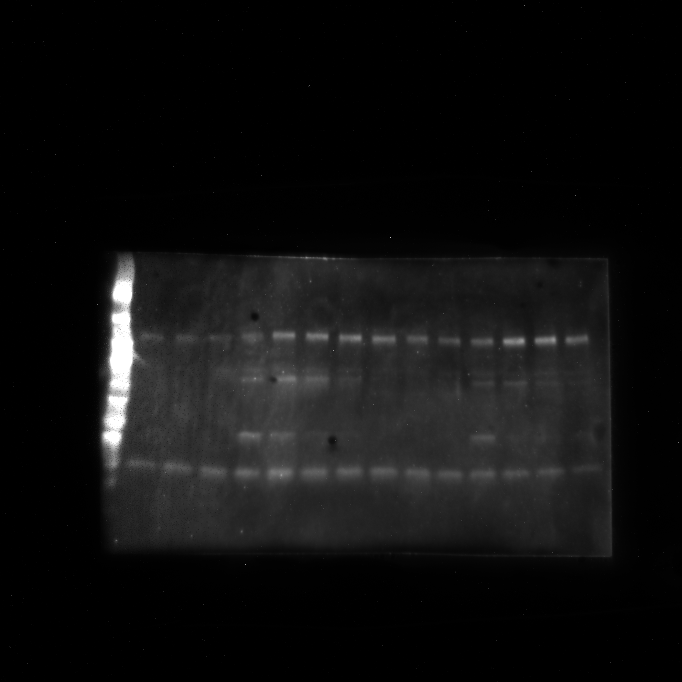

Supplement: Figure 6—figure supplement 2—source data 1. [file elife-83561-fig6-figsupp2-data1.zip › Figure 6-figure supplement 2-source data 1 A p-IkBa.tif]

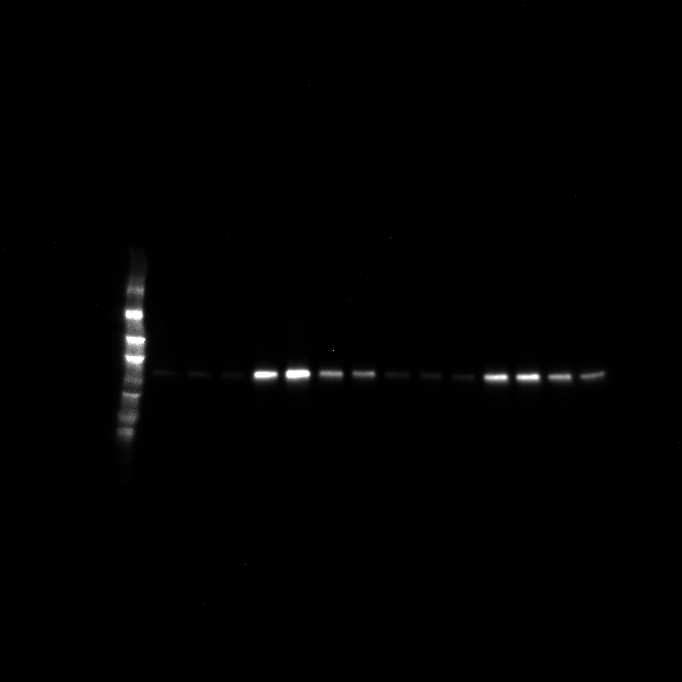

Supplement: Figure 6—figure supplement 2—source data 1. [file elife-83561-fig6-figsupp2-data1.zip › Figure 6-figure supplement 2-source data 1 A p-p65.tif]

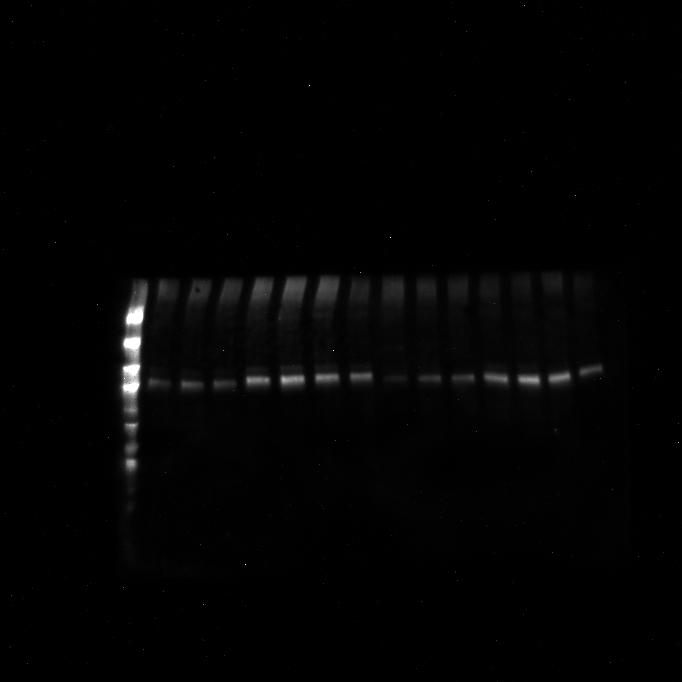

Supplement: Figure 6—figure supplement 2—source data 1. [file elife-83561-fig6-figsupp2-data1.zip › Figure 6-figure supplement 2-source data 1 A p-PKC.tif]

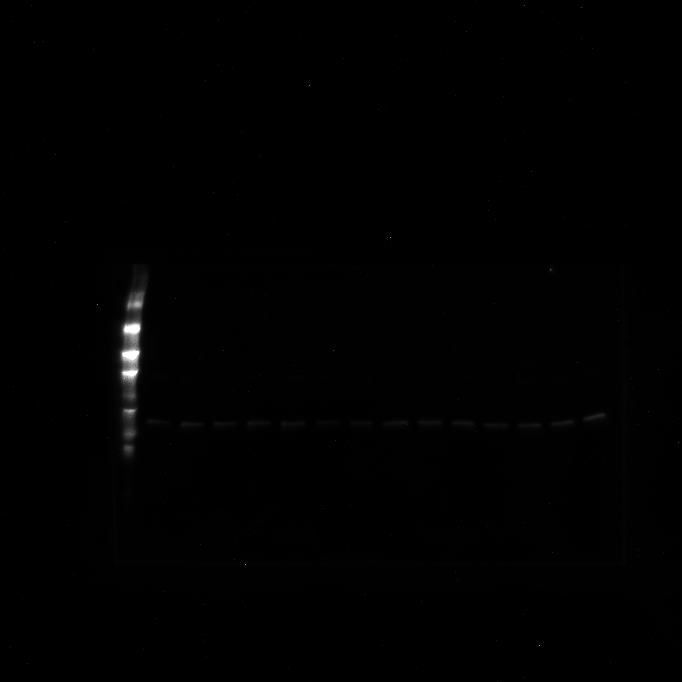

Supplement: Figure 6—figure supplement 2—source data 1. [file elife-83561-fig6-figsupp2-data1.zip › Figure 6-figure supplement 2-source data 1 A p-STAT5.tif]

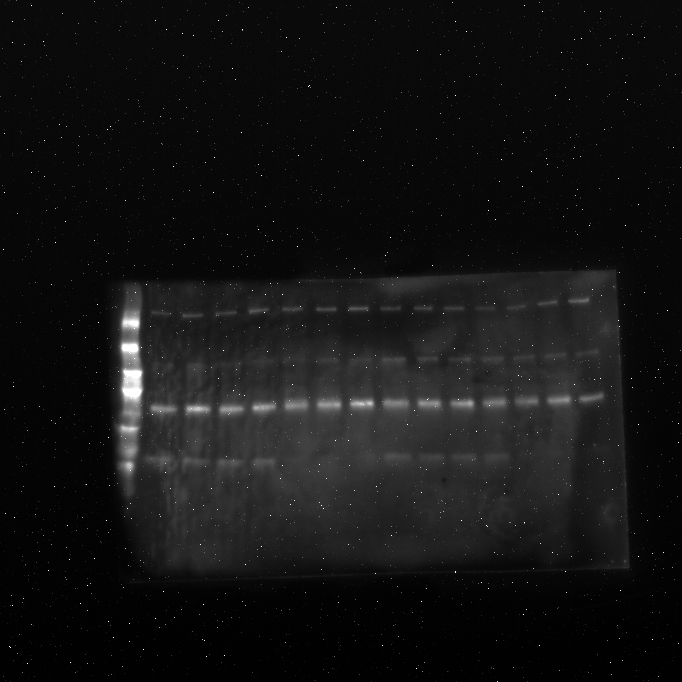

Supplement: Figure 6—figure supplement 2—source data 1. [file elife-83561-fig6-figsupp2-data1.zip › Figure 6-figure supplement 2-source data 1 A total IkBa.tif]

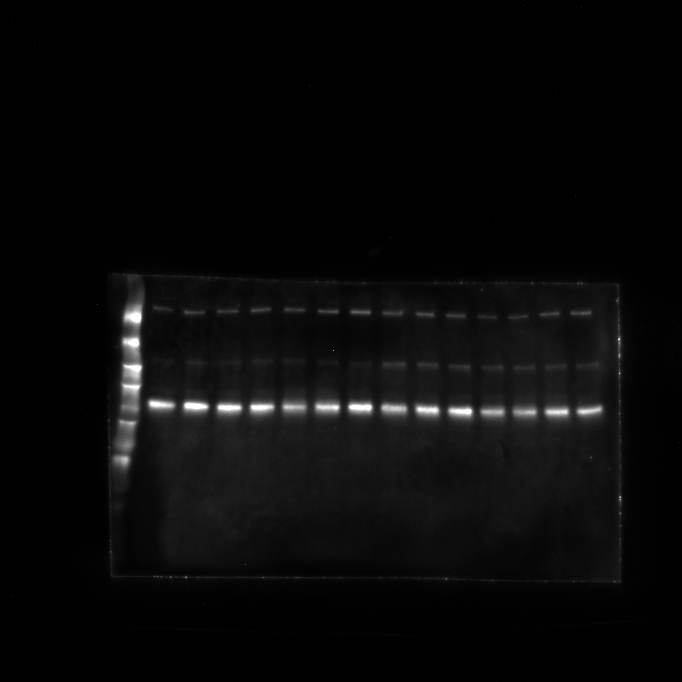

Supplement: Figure 6—figure supplement 2—source data 1. [file elife-83561-fig6-figsupp2-data1.zip › Figure 6-figure supplement 2-source data 1 A total p65.tif]

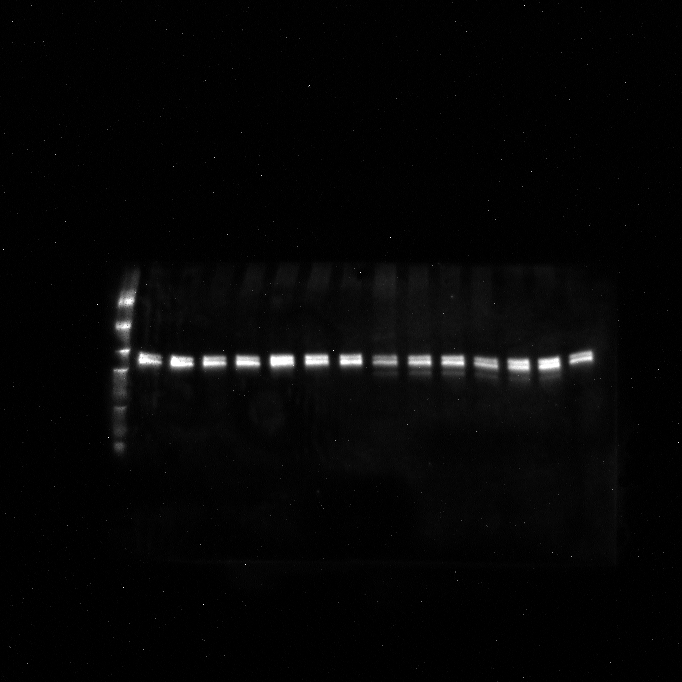

Supplement: Figure 6—figure supplement 2—source data 1. [file elife-83561-fig6-figsupp2-data1.zip › Figure 6-figure supplement 2-source data 1 A total STAT5.tif]

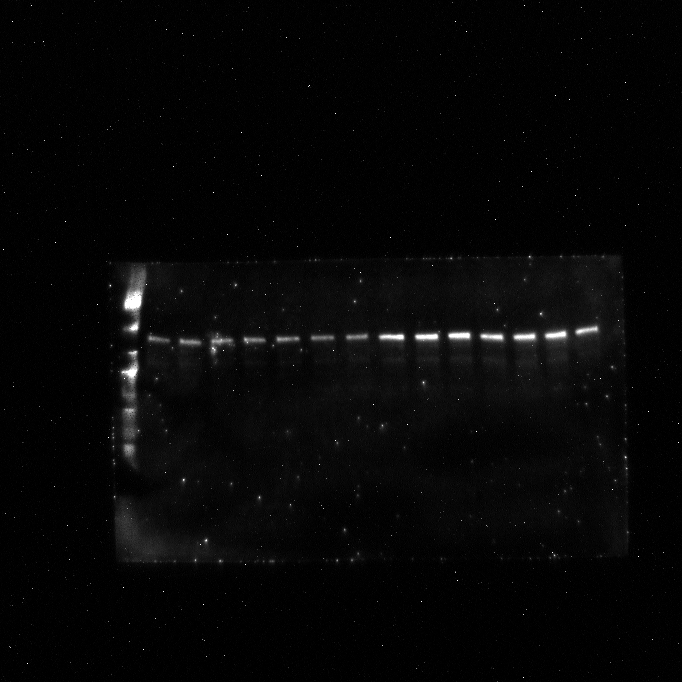

Supplement: Figure 6—figure supplement 2—source data 1. [file elife-83561-fig6-figsupp2-data1.zip › Figure 6-figure supplement 2-source data 1 A total-FAK.tif]

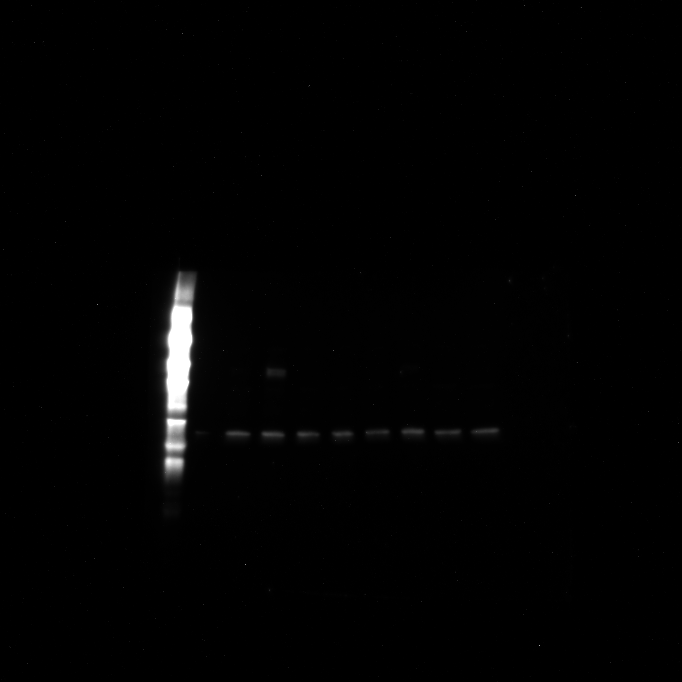

Supplement: Figure 6—figure supplement 2—source data 1. [file elife-83561-fig6-figsupp2-data1.zip › Figure 6-figure supplement 2-source data 1 B p-STAT5.tif]

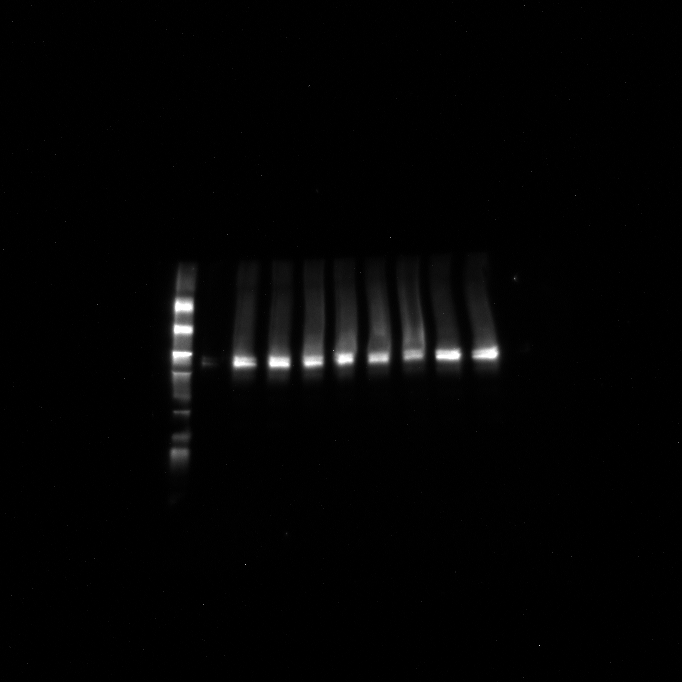

Supplement: Figure 6—figure supplement 2—source data 1. [file elife-83561-fig6-figsupp2-data1.zip › Figure 6-figure supplement 2-source data 1 B total STAT5.tif]

Figure 6-figure supplement 2-source data 1

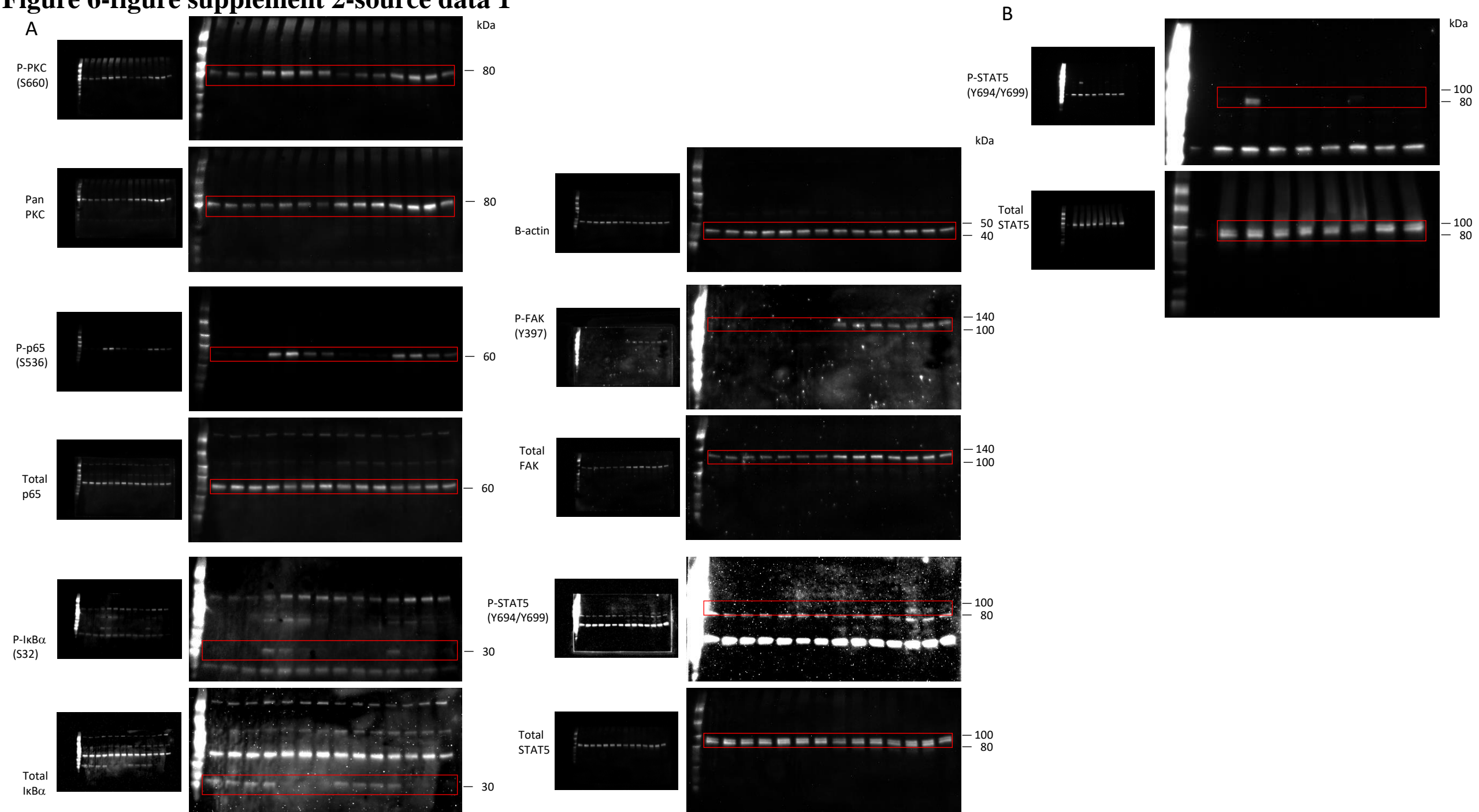

Supplement: Figure 6—figure supplement 2—source data 2. [file elife-83561-fig6-figsupp2-data2.pdf]
